# Proper splicing contributes to visual function in the aging *Drosophila *eye

**DOI:** 10.1111/acel.12817

**Published:** 2018-07-12

**Authors:** Rachel Stegeman, Hana Hall, Spencer E. Escobedo, Henry C. Chang, Vikki M. Weake

**Affiliations:** ^1^ Department of Biochemistry Purdue University West Lafayette Indiana; ^2^ Department of Biological Sciences Purdue University West Lafayette Indiana; ^3^ Purdue University Center for Cancer Research Purdue University West Lafayette Indiana; ^4^ Present address: University of Minnesota Medical School Minneapolis Minnesota

**Keywords:** aging, *Drosophila*, eye, photoreceptor, splicing, vision

## Abstract

Changes in splicing patterns are a characteristic of the aging transcriptome; however, it is unclear whether these age‐related changes in splicing facilitate the progressive functional decline that defines aging. In *Drosophila*, visual behavior declines with age and correlates with altered gene expression in photoreceptors, including downregulation of genes encoding splicing factors. Here, we characterized the significance of these age‐regulated splicing‐associated genes in both splicing and visual function. To do this, we identified differential splicing events in either the entire eye or photoreceptors of young and old flies. Intriguingly, aging photoreceptors show differential splicing of a large number of visual function genes. In addition, as shown previously for aging photoreceptors, aging eyes showed increased accumulation of circular RNAs, which result from noncanonical splicing events. To test whether proper splicing was necessary for visual behavior, we knocked down age‐regulated splicing factors in photoreceptors in young flies and examined phototaxis. Notably, many of the age‐regulated splicing factors tested were necessary for proper visual behavior. In addition, knockdown of individual splicing factors resulted in changes in both alternative splicing at age‐spliced genes and increased accumulation of circular RNAs. Together, these data suggest that cumulative decreases in splicing factor expression could contribute to the differential splicing, circular RNA accumulation, and defective visual behavior observed in aging photoreceptors.

## 1 INTRODUCTION

Normal aging is characterized by progressive and time‐dependent functional decline (Kenyon, 2010). Recent studies show that changes in splicing are part of the transcriptional signature of aging cells (Deschênes & Chabot, 2017; Stegeman & Weake, 2017). Age‐associated changes in alternative splicing have been observed in human brain and in mouse skin, skeletal muscle, bone, thymus, and white adipose tissue (Rodríguez et al., 2016; Tollervey et al., 2011). Splicing is a highly regulated and integral part of proper gene expression; thus, slight perturbations in splicing can lead to altered function and/or expression. Despite the correlation between splicing and aging in many studies, it is unclear whether age‐associated changes in splicing contribute to the functional decline observed during normal aging.

Splicing is catalyzed by the spliceosome and its associated proteins, which are targeted to splice junctions via splicing factors. More than 300 proteins and several noncoding RNAs are involved in splicing, providing multiple opportunities for regulation (Jurica & Moore, 2003). Alternative splicing, the differential use of exons to produce distinct transcripts occurs at over 90% and 60% of protein‐coding genes in humans and *Drosophila *respectively (Graveley et al., 2011; Wang et al., 2008). The binding of splicing factors to elements within the nascent transcript largely governs the differential inclusion of exons, thereby determining the splicing outcome of a particular transcript (Matera & Wang, 2014). Knockdown of individual splicing factors in cultured *Drosophila *cells revealed that half of all splicing events are regulated by more than one splicing factor, illustrating the complicated and combinatorial effect of splicing factors in determining splice‐site usage (Brooks et al., 2015).

Many splicing factors show age‐related changes in their expression that correlate with changes in alternative splicing during aging (Harries et al., 2011; Lee et al., 2016; Mazin et al., 2013; Rodríguez et al., 2016; Tollervey et al., 2011; Wood, Craig, Li, Merry, & Magalhães, 2013). For example, as many as one‐third of all splicing factors exhibit altered expression with age in human blood (Holly et al., 2013). Despite this correlation between splicing factor levels and splicing outcomes during aging, it is not clear whether the decreased expression of splicing factors causes the observed age‐associated changes in splicing. As several splicing factors are mutated in age‐related eye disease, understanding the link between individual splicing regulators and age‐associated changes in splicing could have important implications for human health (Daiger, Sullivan, & Bowne, 2013; Sullivan et al., 2006; Van Cauwenbergh et al., 2017).

As in other organisms and tissues, aging *Drosophila* photoreceptors show altered expression of many genes involved in RNA processing and splicing (Hall et al., 2017). We previously showed that the altered gene expression profile of aging photoreceptors correlates with a decline in visual behavior, termed visual senescence (Carbone et al., 2016; Hall et al., 2017). As other transcriptome studies have also shown a correlation between splicing factor expression and splicing during aging (Stegeman & Weake, 2017), we wondered whether age‐associated alterations in splicing could contribute to visual senescence in flies. To examine this question, we sought to characterize the contribution of individual age‐regulated splicing factors to age‐associated changes in alternative splicing and visual function in the *Drosophila* eye. Here, we identified changes in splicing in both photoreceptors and eyes, and show that age‐regulated changes in splicing occur predominantly in genes with cell‐type‐specific functions. In addition, we find that many splicing factors are required both for proper visual function, and for splicing of a subset of target transcripts. Moreover, knockdown of splicing factors impacts both alternative splicing events and noncanonical splicing outcomes such as circular RNA (circRNA) production. Together, our data suggest that decreased levels of splicing factors could potentially contribute to both age‐associated splicing defects and visual senescence.

## 2 RESULTS

### 2.1 Age‐downregulated splicing factors are necessary for visual behavior in young flies

**Figure 1:**
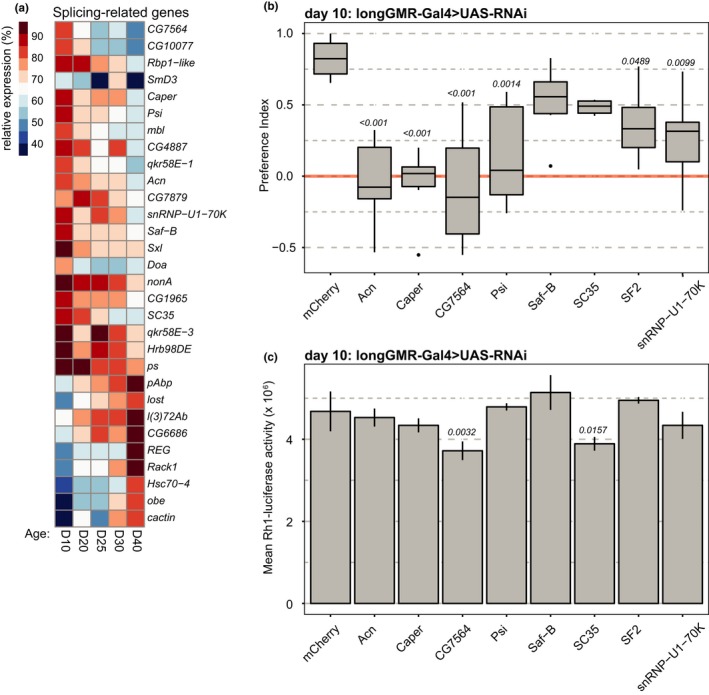
Age‐downregulated splicing factors are necessary for visual behavior in young flies. (a) Heatmap showing expression of genes with a splicing‐related Gene Ontology (GO) term (*RNA splicing*, GO:0008380) that were also significantly differentially expressed between day 10 and day 40 in photoreceptors. Mean expression is shown for the indicated days post eclosion as a percentage of the maximum expression level. (b) Box plots showing preference indices for positive phototaxis in 10‐day‐old male flies expressing RNAi against the indicated gene in the eye under control of *longGMR‐Gal4* (*n* = 6 experiments; 21–34 flies/experiment). *p*‐value, Dunnett's test vs. *mCherry* control. (c) Bar plots showing luciferase activity in 10‐day‐old male flies expressing RNAi against the indicated gene in the eye under control of *longGMR‐Gal4*. Luciferase was expressed in photoreceptors under control of the *Rh1* promoter (*Rh1‐luc*) as an indicator of photoreceptor viability (*n* = 3 experiments; 2 heads/experiment). *p*‐value, Dunnett's test vs. *mCherry* control

Previously, we showed that genes involved in splicing have altered expression in aging *Drosophila* photoreceptors (Hall et al., 2017). Here, we sought to determine whether decreased levels of individual splicing factors in aging photoreceptors could contribute to the decline in visual function with age. To do this, we first identified genes that were significantly differentially expressed in photoreceptors between days 10 and 40 using DESeq2 (adjusted *p*‐value < 0.005) and compared these with genes that had the Gene Ontology (GO) term “RNA splicing”; 93% of male flies survived until day 40 under our experimental conditions, with a maximal lifespan of 80–85 days (Hall et al., 2017). We identified 30 genes involved in RNA splicing that were differentially expressed between day 10 and 40 in photoreceptors (Figure 1a, Supporting Information Table S1). Two‐thirds of these splicing‐related genes were downregulated during aging including splicing factors such as *SC35* and *Caper* that have been previously shown to play a role in alternative splicing in neurons (Gabut, Dejardin, Tazi, & Soret, 2007; Olesnicky, Bono, Bell, Schachtner, & Lybecker, 2017). Based on this analysis, we identified seven splicing‐related genes that showed consistent patterns of decline with age, had relatively strong fold decreases during aging, and had available RNAi fly lines for functional analysis. These genes include the splicing factors *Acn*, *CG7564* (human LUC7‐like protein homolog), *SC35, Caper*, and *Saf‐B*. In addition, we selected a core spliceosomal component of the U1 snRNP, *snRNP‐U1–70K*, and *Psi*, which associates with the U1‐snRNP and has been shown to play a role in courtship behavior in flies (Wang et al., 2016). We also selected *SF2/dASF* (*SRSF1*) for functional analysis because SF2 has photoreceptor‐specific gene targets and has been implicated in aging (Blanco & Bernabeu, 2011, 2012; Gabut et al., 2007; Harries et al., 2011); however, *SF2* expression was not significantly downregulated in aging photoreceptors in our analysis.

To test whether these splicing factors were necessary for proper visual function, we expressed single copy *VALIUM20* RNAi transgenes against each candidate gene in the eye using the *longGMR‐Gal4 *driver (Wernet et al., 2003). We then performed phototaxis assays using a T‐maze apparatus, in which flies were given a choice between light (positive phototaxis, preference index 1.0) and dark (negative phototaxis, preference index −1.0). We previously showed that male flies have a significant decrease in phototactic behavior between days 10 and 40, indicative of reduced visual function (Hall et al., 2017). Because both phototaxis and visual senescence differ between male and female flies, and because the original transcriptome profiling was performed in male flies, here we examined phototaxis in young male flies expressing RNAi against each candidate gene. Young flies expressing RNAi against *mCherry* under control of the *longGMR‐Gal4* driver showed strong positive phototaxis at 10 days post eclosion (Figure 1b). In contrast, expression of RNAi against six of the eight splicing factors tested resulted in significant decreases in phototaxis relative to the *mCherry* RNAi control (Figure 1b). One possible explanation could be that proper expression of splicing factors is required for photoreceptor viability. To test if knockdown of these splicing factors caused retinal degeneration, we used a luciferase reporter under control of the *ninaE* (*Rh1*) promoter (*Rh1‐luc*) and optical neutralization to assess rhabdomere integrity (Franceschini & Kirschfeld, 1971). Although *CG7564 *and *SC35 *showed modest decreases in *Rh1‐luc *activity relative to the control (Figure 1c), we could only detect very modest retinal degeneration in *CG7564* by optical neutralization (Supporting Information Figure S1). Moreover, *SC35* showed modest, but not significant decreased phototaxis relative to the control (Figure 1b), suggesting that any retinal degeneration was not sufficient to significantly reduce visual behavior.

### 2.2 Differential splicing events occur in the aging *Drosophila* eye

As the age‐downregulated splicing factors were necessary for visual behavior in young flies, we next asked whether the eye showed changes in splicing during aging. To do this, we examined splicing in dissected eyes from male *Rh1‐Gal4>KASH‐GFP* flies, the same genotype previously used for photoreceptor‐specific transcriptome profiling (Hall et al., 2017). We performed RNA‐seq on dissected eyes from flies collected at 10 and 40 days post eclosion and obtained 38–52 million high‐quality paired‐end reads for each biological replicate (*n* = 3). Similar numbers of reads (average of 32 million) were previously obtained for photoreceptors (Hall et al., 2017). We then analyzed alternative splicing events between days 10 and 40 in eyes using JunctionSeq, which identifies differential splicing events, defined as exon and/or splice junction usage including novel junctions, relative to the overall expression of the gene (Hartley & Mullikin, 2016).

**Figure 2:**
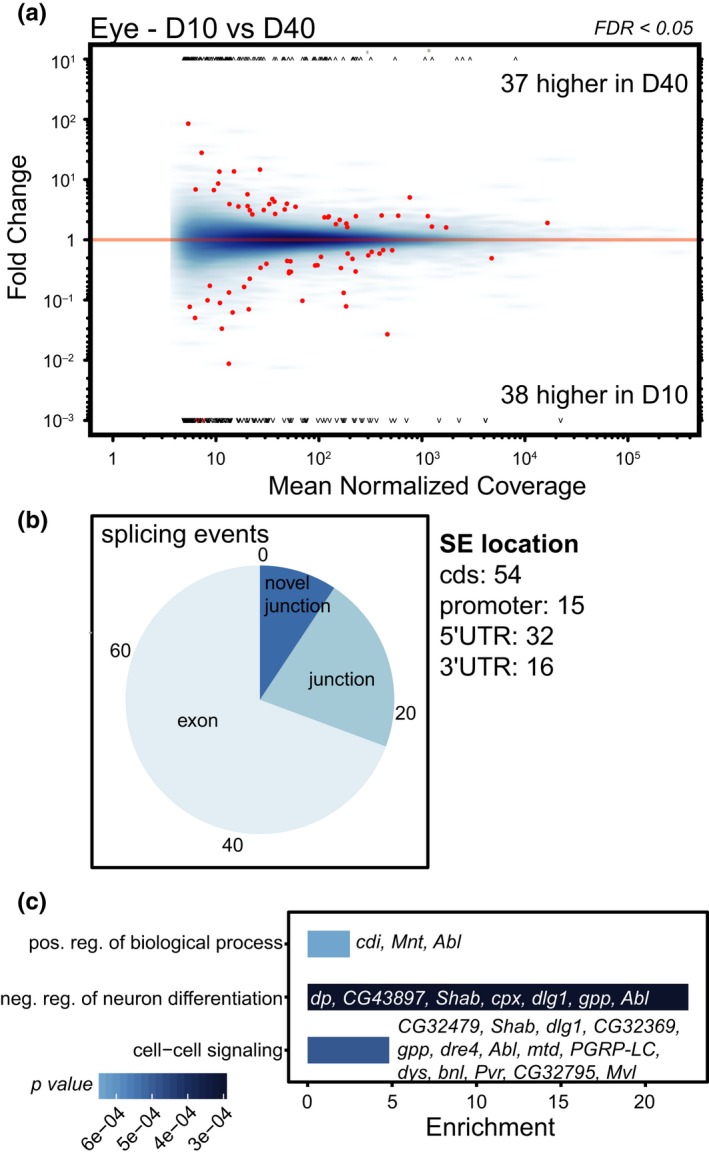
Differential splicing events occur in the aging *Drosophila *eye. (a) MA plots showing differential exon or junction usage in day 10 compared to day 40 eyes plotted as the fold change (*y*‐axis) for each splicing event as a function of the overall expression of the gene (mean normalized coverage, *x*‐axis). Significant differential splicing events (false discovery rate, FDR < 0.05) are shown in red relative to all splicing events (blue). (b) Pie chart showing the proportion of age‐regulated splicing events (SE) including differential exon (52) or junction usage (16 known; seven novel). The number of SEs mapping to regions of each gene is shown in the right panel, with some SEs mapping to overlapping regions (cds, coding sequence; UTR, untranslated region). (c) Bar plot displaying significantly enriched GO terms for genes that show differential splicing with age relative to all genes that are expressed in the eye. Enrichment *p*‐values not corrected for multiple testing. The genes that are differentially spliced with age for a particular GO term are shown within or beside each bar

Using this approach, we identified 75 significant differential splicing events (FDR < 0.05) corresponding to 48 genes in eyes between days 10 and 40 (Figure 2a, Supporting Information Table S2). There were similar numbers of differential splicing events that showed increased or decreased abundance with age in the eye, including differential exon or splice junction usage (Figure 2b). The differential splicing events detected mapped predominantly to the coding regions of genes, suggesting that these would be likely to affect the relative abundance of protein isoforms during aging (Figure 2b). However, we also detected alternative transcription start site usage and differential splicing events in both the 5′ and 3′ untranslated regions, which could represent alternative promoter usage and/or polyadenylation. The genes that were differentially spliced with age in the eye were enriched for somewhat broad GO categories including negative regulation of neuron differentiation and cell–cell signaling and include genes that have previously been shown to play a role in visual function in flies including *discs large 1* *(dlg1)*, *branchless* *(bnl), *and *Na/Ca‐exchange protein* (*Calx*; Mendoza‐Topaz et al., 2008; Mukherjee, Choi, & Banerjee, 2012; Wang et al., 2005). The *Drosophila *compound eye contains a number of cell types in addition to photoreceptors including additional structural and pigment cells (Ready, Hanson, & Benzer, 1976). Thus, we wondered whether differential splicing events in photoreceptors might be masked by the cellular heterogeneity present in the dissected eye tissue.

### 2.3 Genes involved in visual function are differentially spliced with age in photoreceptors

**Figure 3:**
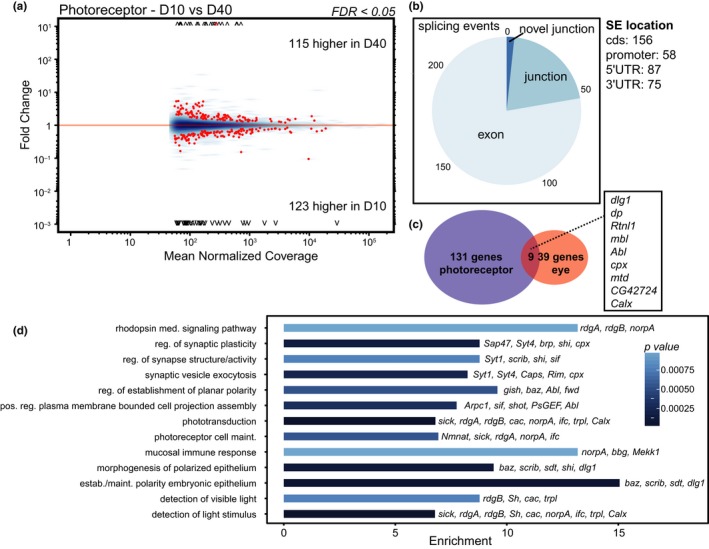
Genes involved in visual function are differentially spliced with age in photoreceptors. (a) MA plots showing differential exon or junction usage in day 10 compared to day 40 photoreceptors (nuclear RNA) as described in Figure 2a. (b) The proportion of age‐regulated splicing events (SE) including differential exon (185) or junction usage (49 known; four novel), and the number of SEs mapping to regions of each gene. (c) Venn diagram showing the number of genes that are differentially spliced with age in eye vs. photoreceptor. Overlapping genes are listed in the box. (d) Bar plot displaying significantly enriched GO terms for differentially spliced genes in photoreceptors.

To identify differential splicing events in aging photoreceptors, we examined our previous RNA‐seq data from affinity‐isolated photoreceptor nuclear RNA from 10‐ and 40‐day‐old *Rh1‐Gal4>KASH‐GFP* flies (Hall et al., 2017). In contrast to the ribo‐depleted cellular RNA from dissected eyes, the photoreceptor RNA‐seq experiment used ribo‐depleted nuclear RNA. We identified a much larger number of differential splicing events using JunctionSeq in aging photoreceptors (238 events/140 genes, FDR < 0.05) compared to the eye (77 events/48 genes; Figure 3a, Supporting Information Table S3). Importantly, we did not observe biases in the number of splicing events detected in more highly expressed genes, indicating that sufficient reads are present in both the photoreceptor and eye RNA‐seq data to identify age‐associated changes in alternative splicing (*compare MA plots*, Figures 2a and 3a). As we identified a higher number of differential splicing events in aging photoreceptors relative to the entire eye, we were concerned that some of these splicing events could be artifacts resulting from analysis of nuclear rather than total cellular RNA. For example, increased pre‐mRNA levels in the nuclear transcriptome could result in increased identification of intron retention events in these data. Differential splice junction usage is an indicator of intron retention because increased levels of retained introns will alter splice junction counts without changing the counts for flanking exons (Hartley & Mullikin, 2016); however, similar proportions of differentially used splice junctions were identified in aging eyes and photoreceptors, respectively (22.9% vs. 20.7%, Figure 3b), indicating that the differential splicing events detected in photoreceptors are unlikely to be due to artifacts resulting from the presence of pre‐mRNA.

Surprisingly, only nine genes were differentially spliced in both the photoreceptor and eye RNA‐seq analysis (Figure 3c). Differentially spliced genes in aging photoreceptors displayed functional enrichment in processes related to visual function including phototransduction, rhodopsin‐mediated signaling pathway, regulation of synaptic plasticity, and photoreceptor cell maintenance (Figure 3d). In the *Drosophila* visual system, light activates the G‐protein‐coupled receptor rhodopsin, leading to a signaling cascade which results in an influx of calcium and depolarization of the photoreceptor neuron, triggering synaptic transmission (Hardie & Juusola, 2015). Several genes differentially spliced with age in photoreceptors encode proteins that are necessary for this phototransduction cascade including *G protein subunit γ at 30A* (*Gγ30A*)*, no receptor potential A* (*norpA*, phospholipase C), *retinal degeneration A* (*rdgA*), and *retinal degeneration B* (*rdgB*; Hardie & Juusola, 2015). In addition, several of the age‐spliced genes encode proteins that are required for synaptic transmission such as *synapse‐associated protein 47 kD* (*Sap47*), *synaptotagmin 1* (*Syt1*), and *complexin* (*cpx*). Thus, age‐related changes in splicing in photoreceptors occur in genes that are required for visual function.

### 2.4 Proper splicing in outer photoreceptors contributes to vision

**Figure 4:**
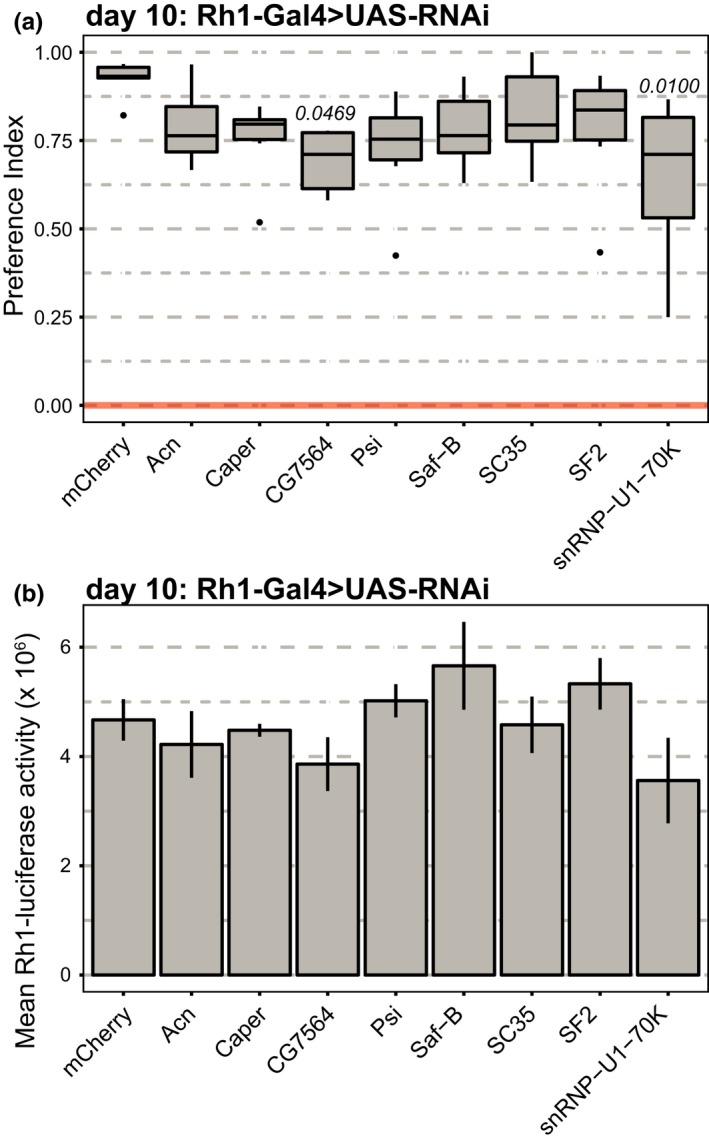
Proper splicing in young photoreceptors contributes to visual behavior but not cell survival. (a) Box plots showing preference indices for phototaxis in 10‐day‐old male flies expressing RNAi under control of *Rh1‐Gal4* (*n* = 6). *p*‐value, Dunnett's test vs. *mCherry* control. (b) Bar plots showing luciferase (*Rh1‐luc*) activity in 10‐day‐old male flies expressing RNAi under control of *Rh1‐Gal4* (*n* = 3 experiments; 2 heads/experiment). *p*‐value, Dunnett's test vs. *mCherry* control.

*Drosophila* have eight photoreceptors in each ommatidium in the compound eye: six outer photoreceptors that express Rh1 and are largely responsible for motion, and two inner photoreceptors that are involved in color vision (Yamaguchi, Desplan, & Heisenberg, 2010). Our splicing analysis in photoreceptors examined the Rh1‐expressing outer photoreceptors, and so we next asked whether proper splicing was also necessary in these outer photoreceptors for visual behavior. We expressed RNAi using *Rh1‐Gal4* (Yoshihara et al., 1999). In contrast, the *longGMR‐Gal4 *is expressed in all photoreceptors (Wernet et al., 2003). Young flies expressing RNAi against *mCherry* in photoreceptors under control of the *Rh1‐Gal4* driver showed strong positive phototaxis at 10 days post eclosion (Figure 4a). While expression of RNAi against all splicing factors resulted in modest decreases in phototaxis relative to the *mCherry *control, only *CG7564* and *snRNP‐U1–70K *significantly reduced phototaxis (Figure 4a). In addition, no retinal degeneration was observed using either the *Rh1‐luc* reporter (Figure 4b) or optical neutralization (Supporting Information Figure S2). Previous studies have shown that the outer R1–R6 photoreceptors are not essential for phototaxis (Yamaguchi et al., 2010), suggesting a possible explanation for the stronger phototaxis phenotype observed with the pan‐photoreceptor *longGMR‐Gal4* driver. Our data tentatively suggest that *CG7564* and *snRNP‐U1–70K* might have a stronger role in regulating splicing of genes necessary for proper visual function.

### 2.5 Age‐regulated splicing factors contribute to proper splicing at age‐spliced genes

**Figure 5:**
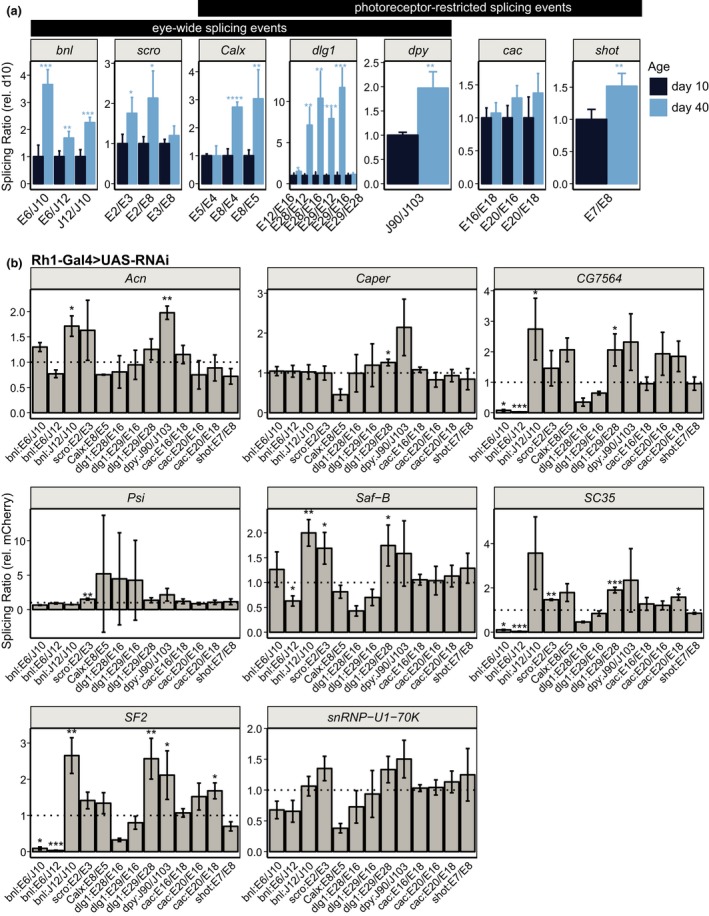
Age‐regulated splicing factors contribute to proper splicing at age‐spliced genes. (a) Bar plots showing qPCR analysis of eye‐wide and photoreceptor‐restricted splicing events in eyes from male flies at day 10 or 40. Relative splicing ratios for the indicated comparisons (E: exon; J: junction), normalized to day 10, which is set to one. *p*‐value, Student's *t* test between days 10 and 40 for indicated splicing event (*n* = 4; *p < 0.05, **p < 0.01, ***p < 0.001). (b) qPCR analysis of splicing events in eyes from 10‐day‐old male flies expressing RNAi against the indicated gene in photoreceptors under control of *Rh1‐Gal4*. Relative splicing ratios for the indicated comparisons, normalized to the *mCherry* control, which is set to one (dotted line). *p*‐value, Student's *t* test between indicated RNAi line and *mCherry* control (not corrected for multiple testing; *n* = 3; *p < 0.05; **p < 0.01, ***p < 0.001)

We and others have identified correlations between reduced expression of splicing‐related genes and altered splicing patterns during aging (Stegeman & Weake, 2017). In *Drosophila *cells, splicing factors act combinatorially to regulate splicing events and more than half of splicing events are regulated by more than one splicing factor (Brooks et al., 2015). Thus, we next asked whether the splicing factors that were necessary for visual function were also required for any of the age‐regulated splicing events. To do this, we first identified a subset of age‐regulated splicing events that could be robustly detected by qPCR analysis in dissected eyes. The genes selected were either differentially spliced with age in the eye (*bnl, scro*), in the photoreceptor (*cac*, *shot*), or in both eyes and photoreceptors (*Calx, dlg1, dpy*; Supporting Information Figure S3, Tables S2 and S3). The splicing events tested included exon skipping (e.g., E18, *cac)*, alternative transcription start site usage (e.g., E5, *Calx*), or transcript isoform switching (e.g*.*, E2, *scro*). Using this approach, we detected statistically significant differences in the ratio of splicing events (exon or junction) within a gene between day 10 and day 40 in dissected eyes for *bnl*, *scro*, *Calx*, *dlg1*, *dpy, *and *shot* (Figure 5a). Some photoreceptor‐specific splicing events, such as E20/E16 for *cac*, were close to being statistically significant (*p* = 0.06) and had relatively high Cq values (28–29 cycles) indicative of low expression, supporting the idea that the cellular heterogeneity in the eye masks detection of some photoreceptor‐specific splicing events. Further supporting this idea, we did not detect statistically significant differential splicing at several of the photoreceptor‐specific events (*CadN*, *dysc*, *Svil, trpl*) by qPCR in dissected eyes, consistent with the RNA‐seq analysis (Supporting Information Figure S4).

Both overall gene expression and levels of specific splicing events can be altered during aging. To test for differences in age‐associated gene expression, rather than splicing, we normalized levels of each splicing event to expression of two external reference genes (*RpL32* and *eIF1A*; Supporting Information Figure S5). These data demonstrate that for most genes tested, the relative levels of individual exons and/or junctions alters with age, independent of changes in overall expression of the gene. However, three of the four photoreceptor‐specific splicing events that we could not detect by qPCR in dissected eyes (*CadN*, *dysc*, *Svil*) correlate with an overall decrease in expression of the gene between day 10 and day 40. Thus, in addition to the cellular heterogeneity in the eye, overall decreases in expression during aging might also mask differential splicing of some genes.

Next, we analyzed these age‐regulated splicing events in eyes from flies expressing RNAi against each of the splicing factors under *Rh1‐Gal4 *control. We used *Rh1‐Gal4* because this did not induce retinal degeneration with any of the splicing factors (Figure 4b, Supporting Information Figure S2) and because its expression begins late during pupal development (Kumar & Ready, 1995). To do this, we expressed RNAi against each splicing factor and examined ratios of splicing events in dissected eyes from 10‐day‐old male flies relative to the *mCherry *control. Using this approach, we identified differential splicing of five of the seven genes examined in at least one of the RNAi lines tested (Figure 5b). Some of these differential splicing events matched the splicing changes observed in the aging eye: for example, expression of RNAi against *Acn*, CG7564, *Saf‐B,* or *SF2 *resulted in an increased ratio of J12/J10 for *bnl* as compared to that of the control, similar to the changes observed between day 10 and day 40 (compare *bnl* J12/J10 in Figure 5a,b). In addition, expression of RNAi against *Psi*, *Saf‐B* or *SC35*, or *Acn* and *SF2*, resulted in similar splicing changes to those observed during aging for *scro *(E2/E3) and *dpy *(J90/J103). However, some of the other splicing changes observed upon splicing factor knockdown were in the opposite direction to the age‐associated splicing events observed. For example, decreased ratios of E6/J10 and E6/J12 for *bnl* were observed in *CG7564*, *SC35,* and *SF2 *knockdowns. In addition, RNAi against some splicing factors resulted in differential splicing events that were not observed during aging, such as differential splicing of E28/E29 for *dlg1* in *Caper*, *CG7564*, *Saf‐B*, *SC35,* and *SF2. *Several splicing factors appear to regulate the same splicing events: In particular, *CG7564*, *SF2* and *SC35* showed similar patterns of splicing changes, as did *Acn* and *Saf‐B*. We did not identify any splicing factors that showed opposing actions on the same splicing event; however, this could be due to the limited number of genes and splicing factors tested. Thus, these data suggest that decreased levels of multiple splicing factors could have both synergistic and/or opposing effects on splicing at a particular gene. Thus, decreased levels of splicing factors in aging photoreceptors could contribute to age‐associated changes in splicing, albeit in a complex interdependent manner.

### 2.6 Knockdown of age‐regulated splicing factors increases circRNA levels

**Figure 6:**
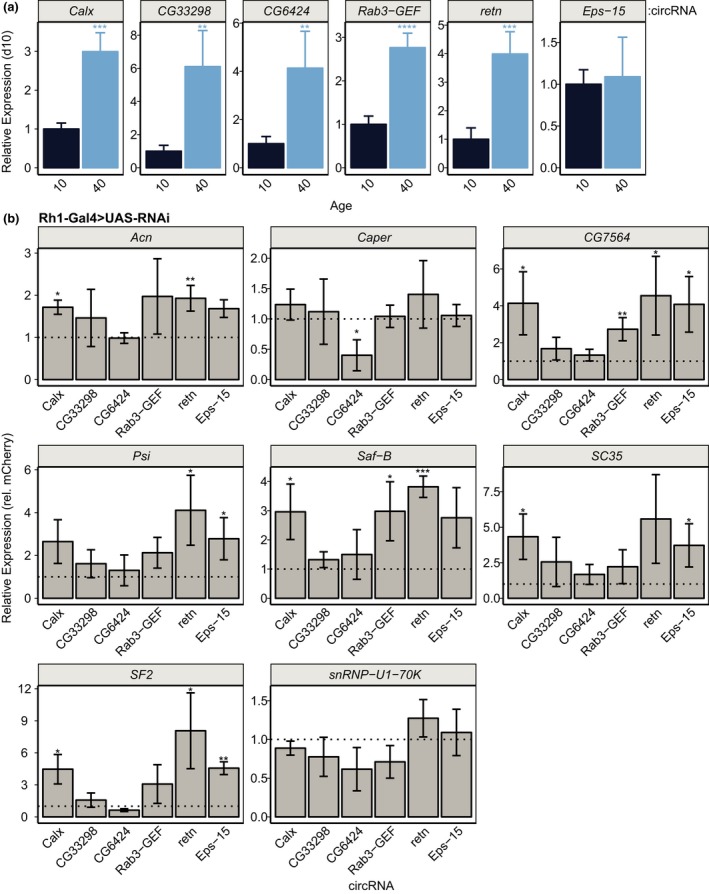
Age‐regulated splicing factors regulate circRNA biogenesis. (a) qPCR analysis of the indicated circRNA levels in eyes from male flies at day 10 or 40. Levels of circRNA were normalized to the reference genes *RpL32* and *eiF1A*, and are shown relative to day 10. *p*‐value, Student's *t* test between days 10 and 40 (*n* = 4; *p < 0.05, **p < 0.01, ***p < 0.001, ****p < 0.0001). (b) qPCR analysis of the indicated circRNA levels in eyes from 10‐day‐old male flies expressing RNAi against the indicated gene in photoreceptors under control of *Rh1‐Gal4*. Levels of circRNA were normalized to the reference genes *RpL32* and *eiF1A*, and are shown relative to the *mCherry* control (dotted line). *p*‐value, Student's *t* test between indicated RNAi line and *mCherry* control (not corrected for multiple testing; *n* = 3; *p < 0.05; **p < 0.01, ***p < 0.001)

In addition to alternative splicing, both the aging brain and *Drosophila *photoreceptors show increased levels of noncanonical splicing events such as circRNA formation (Gruner, Cortes‐Lopez, Cooper, Bauer, & Miura, 2016; Hall et al., 2017; Westholm et al., 2014). CircRNAs result from back‐splicing events from protein‐coding genes and can accumulate to much higher levels than their associated linear transcript (Wilusz, 2017). Recent studies have shown that depletion of spliceosomal components in *Drosophila *cells increases levels of circRNAs, while reducing levels of their associated linear mRNA (Liang et al., 2017). We previously showed that aging *Drosophila *photoreceptors accumulate circRNAs (Hall et al., 2017), but it is unclear whether increased biogenesis or accumulation due to the high stability of circRNAs underlies this age‐dependent increase. As in photoreceptors, we detected increased levels of circRNAs in 40‐day‐old eyes relative to 10‐day‐old eyes using qPCR (Figure 6a). To test whether decreased levels of splicing factors affected the relative abundance of these circRNAs, we examined their levels in eyes from flies expressing photoreceptor‐specific RNAi against each splicing factor under *Rh1‐Gal4 *control (Figure 6b). Similar to the observations reported by Liang et al. (2017), 16/17 significant changes in circular RNA levels in the eyes upon splicing factor knockdown involved increased circRNA levels (Figure 6b). Only RNAi against *Caper* reduced levels of *CG6424* circRNA relative to the *mCherry* control. We conclude that age‐associated decreases in expression of splicing‐related genes could affect both alternative splicing and circRNA biogenesis. As these splicing factors are broadly required for proper visual function in young flies, our data support a model in which proper splicing could contribute to maintaining photoreceptor function in the aging eye.

## 3 DISCUSSION

In this study, we identified age‐regulated splicing events in the eye and photoreceptor neurons of *Drosophila*. Age‐regulated changes in splicing were observed in genes involved in visual function in photoreceptors, but not in the eye, suggesting that cell‐type‐specific transcriptome studies reveal changes in splicing that may be masked in whole tissue RNA‐seq analysis. We demonstrate that most of the splicing‐related genes that were downregulated with age are necessary for proper visual behavior. Knockdown of these splicing factors results in altered patterns of splicing that partially resemble those observed during aging, including both changes in alternative splicing and in production of noncanonical RNAs such as circRNAs. Our data demonstrate that proper splicing, which requires the coordinated activity of many different splicing factors and additional proteins, is necessary for visual function. Moreover, our observations suggest that age‐associated reductions in the expression of individual splicing factors might perturb proper splicing and thereby contribute to visual senescence.

One surprising outcome from this study was the low overlap in the differentially spliced genes identified between aging photoreceptors and eyes. With some limited exceptions (*shot*), the qPCR analysis in independent eye samples largely confirms this result, suggesting that cell‐type specific changes in splicing are difficult to detect in whole tissue samples. These differences in detection do not reflect an overall lack of coverage for the eye RNA‐seq data, which had higher numbers of average reads compared to the photoreceptor data. Rather, we attribute these differences to two major factors: (a) cellular heterogeneity; and (b) nuclear vs. cellular RNA analysis. If a gene is broadly expressed in different cell types within a tissue, but an exon is only differentially used within a subpopulation of cells, substantially higher number of reads would be required to detect a splicing event when examining RNA from the whole tissue. Further, if opposing splicing events occur in two different cell types within a tissue, RNA isolated from the tissue would represent an average of these events. In addition, low read coverage for genes that are expressed at relatively low levels, albeit specifically within a specific cell type, reduces the statistical power necessary to detect differential splicing events. Lastly, some alternative splicing events result in mis‐spliced transcripts that are targeted for nonsense‐mediated decay: These differential splicing events would be detected in nuclear RNA, but not in total cellular RNA due to their rapid degradation. We note that in contrast to photoreceptors, we did not observe decreased expression of most of the splicing‐related genes in aging eyes (Supporting Information Table S4). We speculate that photoreceptor neurons might be more vulnerable to age‐associated decreases in splicing factor expression, and consequently altered splicing, than other cell types in the eye.

While our data demonstrate that proper splicing is necessary for visual function, it also raises the question of whether changes in alternative splicing or increased biogenesis of noncanonical circRNAs drive visual senescence. Could the genes with altered splicing patterns or increased circRNA levels in aging photoreceptors contribute to visual senescence? A number of splice‐site mutations have been identified in age‐related diseases including age‐related macular degeneration and Hutchinson‐Gilford progeria (Allikmets et al., 1997; De Sandre‐Giovannoli et al., 2003). In addition, expression of splicing factors has been shown to directly modulate senescence and lifespan (Harries et al., 2011; Heintz et al., 2017; Holly et al., 2013; Tang et al., 2013). Here, we find that seven of the genes with altered splicing in aging *Drosophila* photoreceptors have predicted human orthologs associated with human eye disease (Supporting Information Table S5). At least one of the genes with altered splicing in aging photoreceptors provides an intriguing candidate for a link between defective splicing and reduced visual function: *dlg1* shows an age‐related change in its splicing profile that would favor expression of a protein product lacking its N‐terminal domain, which is necessary for visual function (Supporting Information Figure S3; Mendoza‐Topaz et al., 2008). Expression of RNAi against *Psi *also increased the proportion of the transcript that lacks the N‐terminal domain, although these changes were not statistically significant due to high variability in the *Psi* qPCR data for this gene (Figure 5b). Potential transcript isoform switching is also observed for genes such as *scro*, *cac,* and *CadN,* but the functional consequences of these changes are unknown. We note that several of the age‐associated splicing changes observed do not seem to represent switches between annotated transcript isoforms, but might instead represent intron retention or mis‐splicing events (e.g., *trpl*, *bnl*, *dysc*; Supporting Information Figure S3).

Several of the circRNAs that accumulate in both aging photoreceptors and in the eye include genes that are required for visual function and/or photoreceptor health. For example, *Calx* encodes a calcium/sodium exchange channel that suppresses retinal degeneration caused by toxic levels of calcium in photoreceptors, a consequence of light stress in flies (Chen et al., 2017; Wang et al., 2005). We observed both differential splicing and circRNA increases at *Calx *(Supporting Information Figure S3). Two recent reports suggest that a subset of circRNAs are translated, suggesting potential regulatory roles during aging (Legnini et al., 2017; Pamudurti et al., 2017). Like the translated circRNAs identified in these studies, the *Calx *circRNA contains a start codon and could be a potential target for translation; however, we have not examined this possibility. As we observe a correlation between visual senescence, alternative splicing, and circRNA biogenesis, we conclude that both types of changes in splicing could contribute to the age‐associated decline in visual behavior.

Tight regulation of the expression of splicing factors is necessary for proper splicing outcomes (Matlin, Clark, & Smith, 2005; Shin & Manley, 2004), and splicing factors themselves exhibit altered patterns of age‐related splicing (Rodríguez et al., 2016). In our data, we observe age‐associated differential splicing of several splicing‐related genes including *muscleblind* (*mbl*) and *Protein on ecdysone puffs* (*Pep)*. Thus, one intriguing possibility suggested by our study is that small perturbations in splicing could progressively cause further changes in splicing, leading to a “snowball” effect. Together, these data suggest that cumulative and progressive changes in the relative levels of spliceosomal components including splicing factors might contribute to chronological aging, especially in long‐lived postmitotic cell types such as neurons.

## 4 EXPERIMENTAL PROCEDURES

See Supporting Information.

## 5 DATA AVAILABILITY

RNA‐seq expression data are accessible through Gene Expression Omnibus (GEO) repository under series accession numbers GSE83431 and GSE106652. Supporting data, scripts, and JunctionSeq output files including graphical splicing representations for significant genes has been deposited at the Purdue University Research Repository (PURR) as a publically available, archived data set and can be accessed using https://doi.org/10.4231/R7ZG6QGD

## CONFLICT OF INTEREST

The authors declare that they have no competing interests.

## AUTHOR CONTRIBUTIONS

RS conceived the study and performed the eye RNA‐seq studies, qPCR, and phototaxis. HH performed the photoreceptor RNA‐seq studies and qPCR analysis. SEE performed phototaxis, luciferase assays, and optical neutralization. HCC generated the Rh1‐luc flies. Data were analyzed by RS and VMW. RS and VMW wrote the manuscript in consultation with the other authors.
