# Supplementary material for: Proper splicing contributes to visual function in the aging Drosophila eye
Source: Aging Cell. 2018 Jul 12;17(5):e12817. doi: 10.1111/acel.12817 (PMC6156539; doi:10.1111/acel.12817)

SUPPLEMENTAL METHODS

Experimental Procedures:

Fly Strains, aging and phototaxis

Flies were raised in 12:12 h light:dark cycle at 25°C on standard fly food. All aged flies were collected on the day of eclosion (day 1) and transferred to fresh vials every 2 - 3 days. All genotypes and genes used in this study are described in Tables S6 and S7 respectively. To generate the mCherry control RNAi flies, a pVALIUM20 plasmid containing a hairpin sequence targeting mCherry (BDSC #35785) was integrated into the attP2 site. To generate *Rh1-Luc* flies, the firefly luciferase coding sequence was cloned into pCaSpeR-ninaE-forward containing the *ninaE* promoter and 3' region (kindly provided by J. O'Tousa) as a *NotI-XbaI* fragment, and transgenic flies were generated. For RNAi knockdowns, *longGMR-Gal4, UAS-RNAi* or *Rh1-Gal4, UAS-RNAi* flies were crossed with TRiP VALIUM20 UAS-RNAi lines in either the attP2 or attP40 sites (Table S5). Phototaxis assays were performed as previously described in (Hall *et al.* 2017) with a choice time of 2 min for *longGMR-Gal4, UAS-RNAi* flies or 30 seconds for *Rh1-Gal4, UAS-RNAi* flies. Briefly, approximately 30 male flies were assayed for the appropriate RNAi cross at 10 days post-eclosion. Six independent phototaxis assays were conducted per RNAi line. Data are presented as box plots of preference indices (positive phototaxis = 1, negative phototaxis = -1) where the lower and upper hinges correspond to the first and third quartiles, and the whiskers extend to the smallest or largest values no more than 1.5 x inter-quartile range from each hinge. Outliers are shown as dots beyond the whiskers. Experimental RNAi lines were compared to the control *mCherry* RNAi using Dunnett's test. A detailed phototaxis protocol is available in the supporting archived data set (see Data Availability section).

Luciferase Assays and Optical Neutralization

Two male heads were homogenized in 100 μL of 1x Cell Culture Lysis buffer (Promega, cat #E1531) and 25 μL of homogenate was added to 50 μL of luciferase reagent (Promega, cat# E1500) in a 96 well plate (Cliniplate, item# 95029770). Two technical samples and three biological (independent crosses) replicates were analyzed for each genotype using a Luminoskan Ascent Luminometer (ThermoFisher, cat #5300172) with 5s plate mixing followed by integrated 10s scans of each well. Optical neutralization was performed as described previously (Franceschini & Kirschfeld 1971) using a Nikon Eclipse 90i wide-field microscope

with Nikon 40x/0.75 Plan Fluor objective and Nikon DS-Ri1 12MP color camera. Detailed protocols are available in the supporting archived data set (see Data Availability section).

qRT-PCR analysis

Eyes (including the lamina) were dissected from 15 adult male flies (2 eyes/fly) using micro-dissection scissors and trimmed to remove excess brain material. RNA was extracted from dissected eyes using Trizol, followed by Zymoprep Direct-zol RNA MicroPrep kit (Zymo Research) and treated with DNAse I. cDNA was generated from 50 ng of RNA using Episcript Reverse Transcriptase (Epicentre) using random hexamer primers. qPCR was conducted using Evagreen 2X Mix (Biotium) and the CFX Connect Real-time system (Biorad). Quantities were determined relative to a serial dilution of cDNA. Primers are described in Table S8. Student's t-test was used to compare splicing ratios between two ages (day 10 and 40), or RNAi lines to the control *mCherry* RNAi (not corrected for multiple testing).

RNA-seq

cDNA libraries were constructed from 25 ng of total nuclear RNA using the NuGEN Ovation RNA seq Systems 1-16 for Model Organism (NuGEN, cat #0350). RNA was DNAse treated, and single-stranded DNA was generated using both random hexamer and oligo-dT primers, and then depleted for ribosomal DNA. The cDNA libraries were ligated to unique adaptors and multiplexed libraries were sequenced with Illumina HiSeq 2500 technology. Paired-end 100 bp reads were sequenced for all aging samples (n = 3).

RNA-seq data analysis

Reads were trimmed using Trimmomatic (v0.36). Quality trimmed reads were mapped to the *D. melanogaster* genome (BDGP6.89) using TopHat (v2.1.1) and exon and splice-junction counts for both known and novel splice junctions was determined using QoRTs (v1.2.42). JunctionSeq analysis (Hartley & Mullikin 2016) was performed to test for differentially used splicing events including both junctions and exons between day 10 and 40. Genes that were differentially expressed in photoreceptors between day 10 and 40 were identified using QoRTs:DESeq2 (padj < 0.1). Wiggle plots generated using QoRTs were visualized using Gviz (v1.22.2).

Functional annotation analysis

GO term enrichment analyses for Biological Process were performed using Gorilla (Eden *et al.* 2009). A background gene set of all expressed genes in either eyes or photoreceptors

respectively was used for comparison for all GO term enrichment. Human homologs of age-spliced genes in *Drosophila* photoreceptors were identified using the DRSC Integrative Ortholog Prediction Tool (DIOPT), and cross-referenced with genes listed on RetNet (https://sph.uth.edu/retnet/) that have been associated with human retinal disease.

Data accessibility statement:

RNA-seq expression data are accessible through Gene Expression Omnibus (GEO) repository under series accession numbers GSE83431 and GSE106652. Supporting data, scripts and JunctionSeq output files including graphical splicing representations for significant genes have been deposited at the Purdue University Research Repository (PURR) as a publically available, archived data set and can be accessed using https://doi.org/10.4231/R7ZG6QGD . Any additional scripts required for analysis are available from the corresponding author on reasonable request.

References:

Eden E, Navon R, Steinfeld I, Lipson D, Yakhini Z (2009). GOrilla: a tool for discovery and visualization of enriched GO terms in ranked gene lists. *BMC Bioinformatics*. **10**, 48.

Franceschini N, Kirschfeld K (1971). [Pseudopupil phenomena in the compound eye of drosophila]. *Kybernetik*. **9**, 159-182.

Hall H, Medina P, Cooper DA, Escobedo SE, Rounds J, Brennan KJ, Vincent C, Miura P, Doerge R, Weake VM (2017). Transcriptome profiling of aging Drosophila photoreceptors reveals gene expression trends that correlate with visual senescence. *BMC Genomics*. **18**, 894.

Hartley SW, Mullikin JC (2016). Detection and visualization of differential splicing in RNA-Seq data with JunctionSeq. *Nucleic Acids Res*. **44**, e127.

SUPPLEMENTAL FIGURE LEGENDS

Figure S1. Expression of RNAi in eyes against 7 of 8 splicing factors does not induce retinal degeneration in 10-day old flies. Optical neutralization was performed on 10-day old male flies expressing RNAi against the indicated gene in eyes under control of *longGMR-Gal4*. Representative images are shown (n = 3).

Figure S2. Expression of RNAi in outer photoreceptors against selected splicing factors does not induce retinal degeneration in 10-day old flies. Optical neutralization was performed on 10-day old male flies expressing RNAi against the indicated gene in outer photoreceptors under control of *Rh1-Gal4*. Representative images are shown (n = 3).

Fig S3. Differential age-associated splicing events identified in photoreceptors or eyes. The position of photoreceptor-restricted (photo.) and eye-wide age-associated splicing events (SE) is shown for a subset of genes that were analyzed by qPCR. Individual genes are shown on different pages and are indicated by the label in the upper left region of the figure. Wiggle plots are shown in the upper two panels representing the relative counts over each gene region for merged day 10 (red) or day 40 (black) photoreceptor (upper panel) or eye (lower panel) RNA-seq data (3 biological replicates). The middle panel shows transcripts present in this gene region: transcripts representing the gene of interest (*eg bnl*, labeled in the top left) have black labels, and co-localizing transcripts for other genes that overlap with the gene of interest are labeled in pink. Transcripts are labeled with the Flybase FBtr ID number. Boxes show exons labeled in red (untranslated regions, UTR) or black (coding sequence, cds), with introns indicated by lines (arrows show transcript direction). The relative position of splicing events and primers used to detect these events by qPCR are shown in the three lower panels. Splicing events that are increased with age (higher in day 40) are indicated by an upward-facing arrow, and splicing events that are decreased with age (lower in day 40) are indicated by a downward-facing arrow. The relative genomic position and chromosomal location are shown at the base of the figure. Alternative graphical representations of splicing for all differentially-spliced genes identified in eyes or photoreceptors (JunctionSeq output) including exon/intron diagrams with marked splicing events and tables are available at https://doi.org/10.4231/R7ZG6QGD .

Fig S4. Several photoreceptor-specific splicing events are not detected in eyes using qPCR. Bar plots showing qPCR analysis of photoreceptor-specific splicing events in eyes from

male flies at day 10 or 40. Relative splicing ratios for the indicated comparisons (E, exon; J, junction), normalized to day 10, which is set to one. No significant difference was identified between ages for the splicing events shown in the eye using Student's *t*-test (n = 4).

Fig S5. Age-associated differences in exon and junction levels reflect differential splicing rather than gene expression. (A) Bar plots showing qPCR analysis of eye- and photoreceptor-specific splicing events in eyes from male flies at day 10 or 40. Levels of each splicing event (E, exon; J, junction) were normalized to the reference genes *RpL32* and *eiF1A* and are shown relative to day 10, which is set to one (n = 4).

Stegeman_FigS1

day 10: longGMR-Gal4>UAS-RNAi

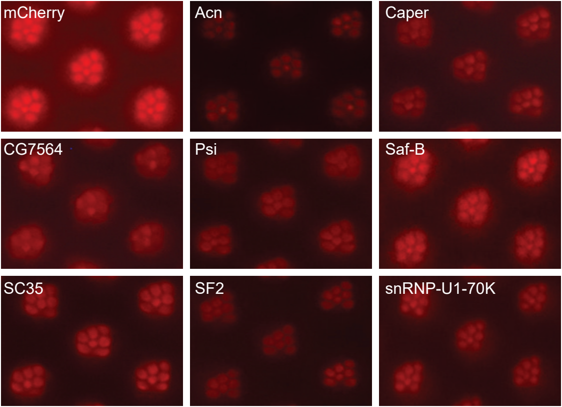

Stegeman_FigS2

day 10: Rh1-Gal4>UAS-RNAi

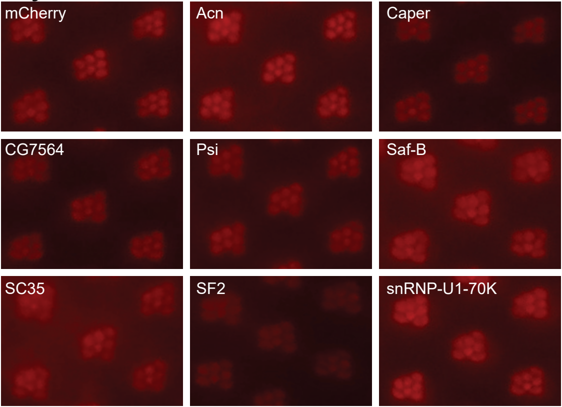

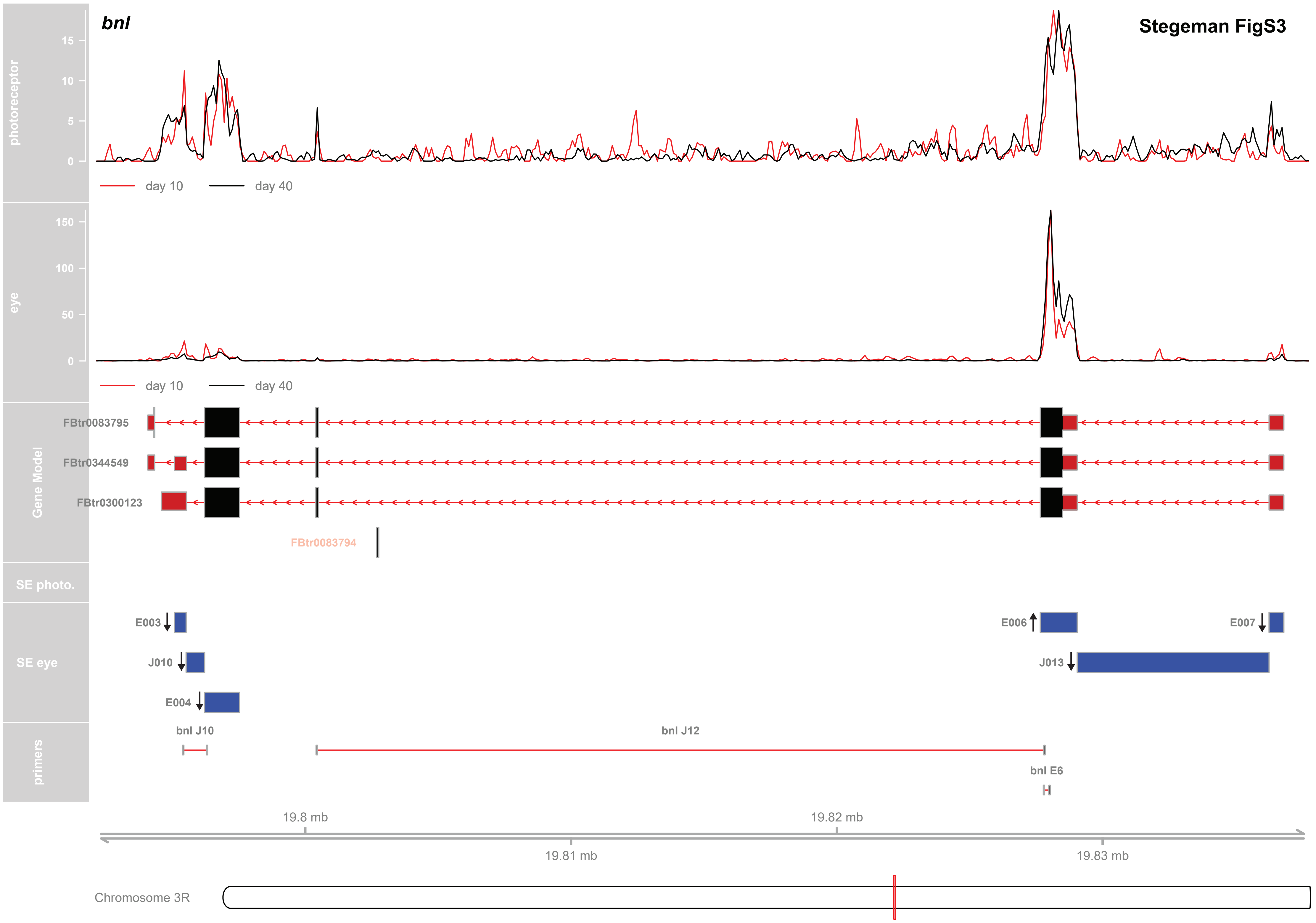

bnl
Stegeman FigS3
photoreceptor
15
10
5
0
day 10
day 40
eye
150
100
50
0
day 10
day 40
Gene Model
FBtr0083795
FBtr0344549
FBtr0300123
FBtr0083794
SE photo.
SE eye
E003
E006
E007
J010
J013
E004
primers
bnl J10
bnl J12
bnl E6
19.8 mb
19.82 mb
19.81 mb
19.83 mb
Chromosome 3R

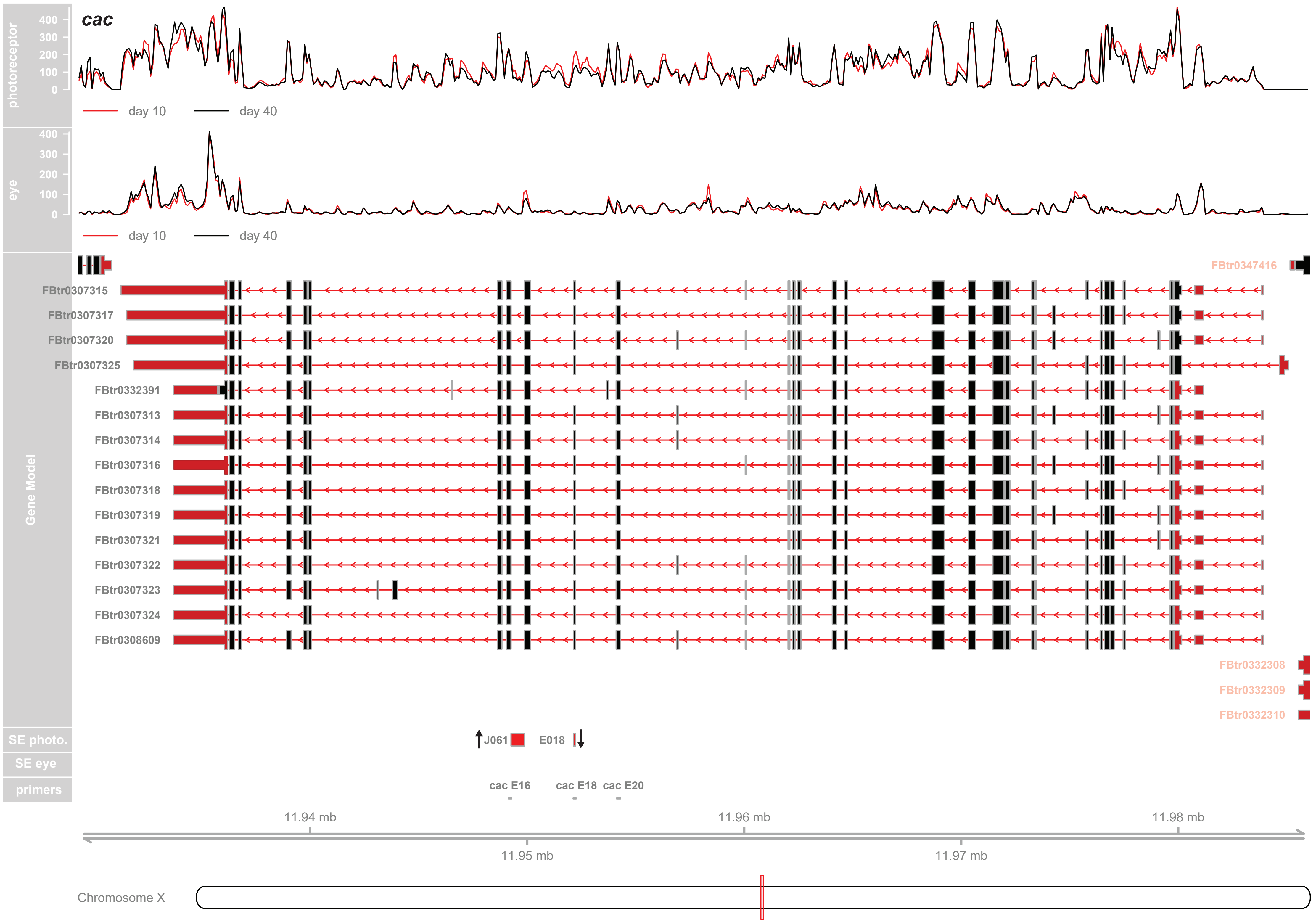
cac
photoreceptor
400
300
200
100
0
day 10
day 40
eye
400
300
200
100
0
day 10
day 40
Gene Model
FBtr0347416
FBtr0307315
FBtr0307317
FBtr0307320
FBtr0307325
FBtr0332391
FBtr0307313
FBtr0307314
FBtr0307316
FBtr0307318
FBtr0307319
FBtr0307321
FBtr0307322
FBtr0307323
FBtr0307324
FBtr0308609
FBtr0332308
FBtr0332309
FBtr0332310
SE photo.
J061
E018
SE eye
primers
cac E16
cac E18
cac E20
11.94 mb
11.96 mb
11.98 mb
11.95 mb
11.97 mb
Chromosome X

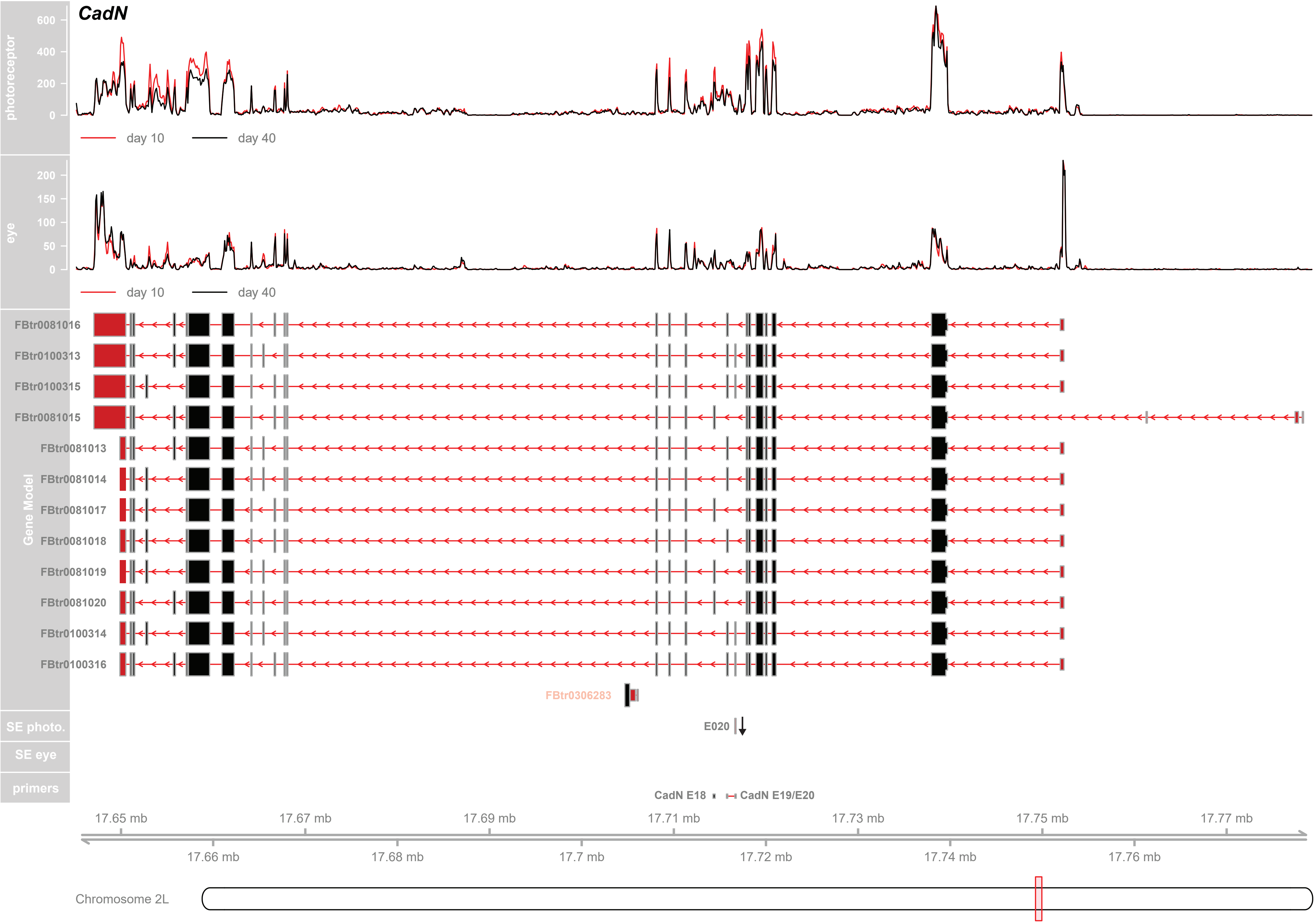
CadN
photoreceptor
600
400
200
0
day 10
day 40
eye
200
150
100
50
0
day 10
day 40
Gene Model
FBtr0081016
FBtr0100313
FBtr0100315
FBtr0081015
FBtr0081013
FBtr0081014
FBtr0081017
FBtr0081018
FBtr0081019
FBtr0081020
FBtr0100314
FBtr0100316
FBtr0306283
SE photo.
E020
SE eye
primers
CadN E18
CadN E19/E20
17.65 mb
17.67 mb
17.69 mb
17.71 mb
17.73 mb
17.75 mb
17.77 mb
17.66 mb
17.68 mb
17.7 mb
17.72 mb
17.74 mb
17.76 mb
Chromosome 2L

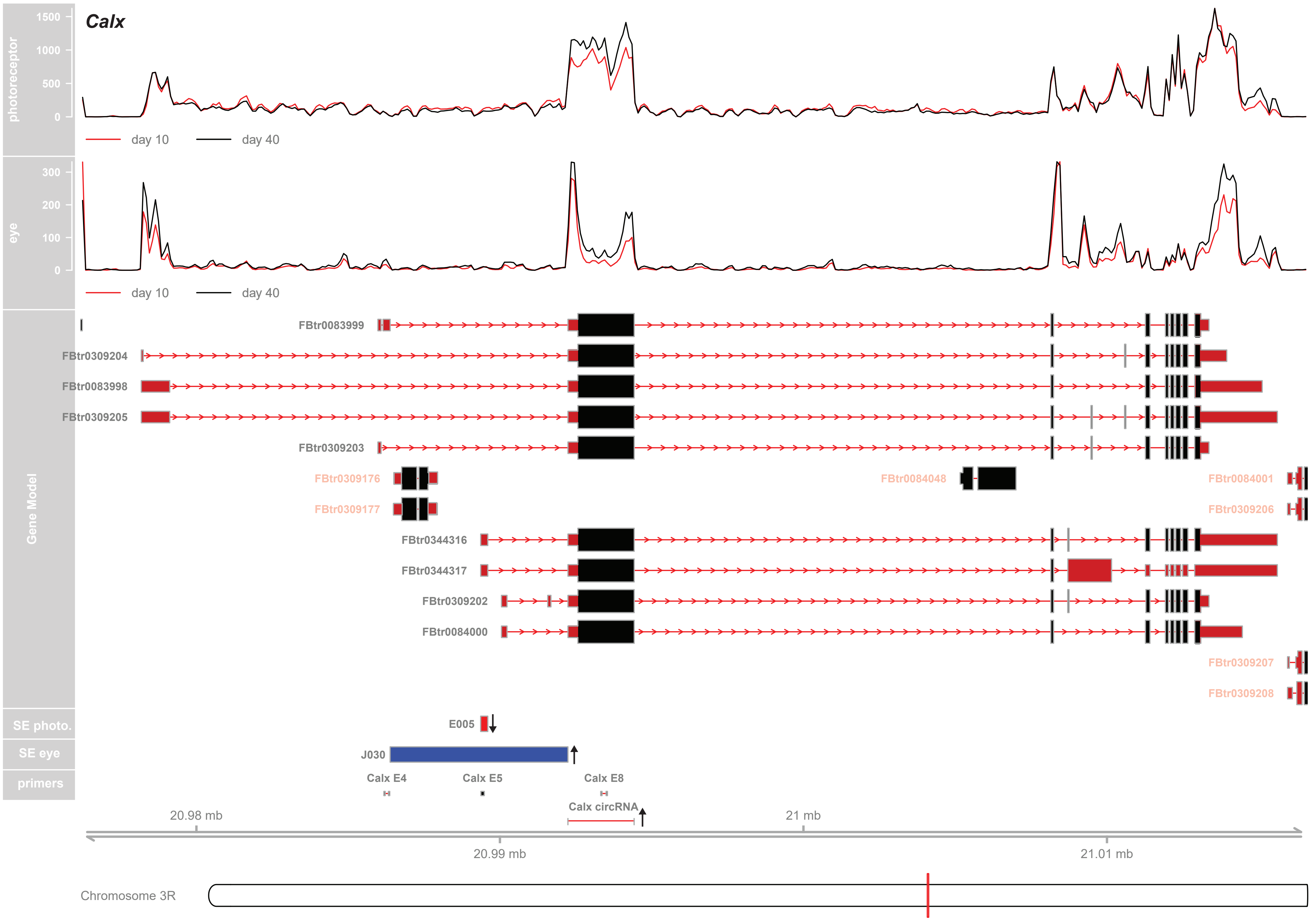

Calx
photoreceptor
1500
1000
500
0
day 10
day 40
eye
300
200
100
0
day 10
day 40
Gene Model
FBtr0083999
FBtr0309204
FBtr0083998
FBtr0309205
FBtr0309203
FBtr0309176
FBtr0084048
FBtr0084001
FBtr0309177
FBtr0309206
FBtr0344316
FBtr0344317
FBtr0309202
FBtr0084000
FBtr0309207
FBtr0309208
SE photo.
E005
SE eye
J030
primers
Calx E4
Calx E5
Calx E8
Calx circRNA
20.98 mb
21 mb
20.99 mb
21.01 mb
Chromosome 3R

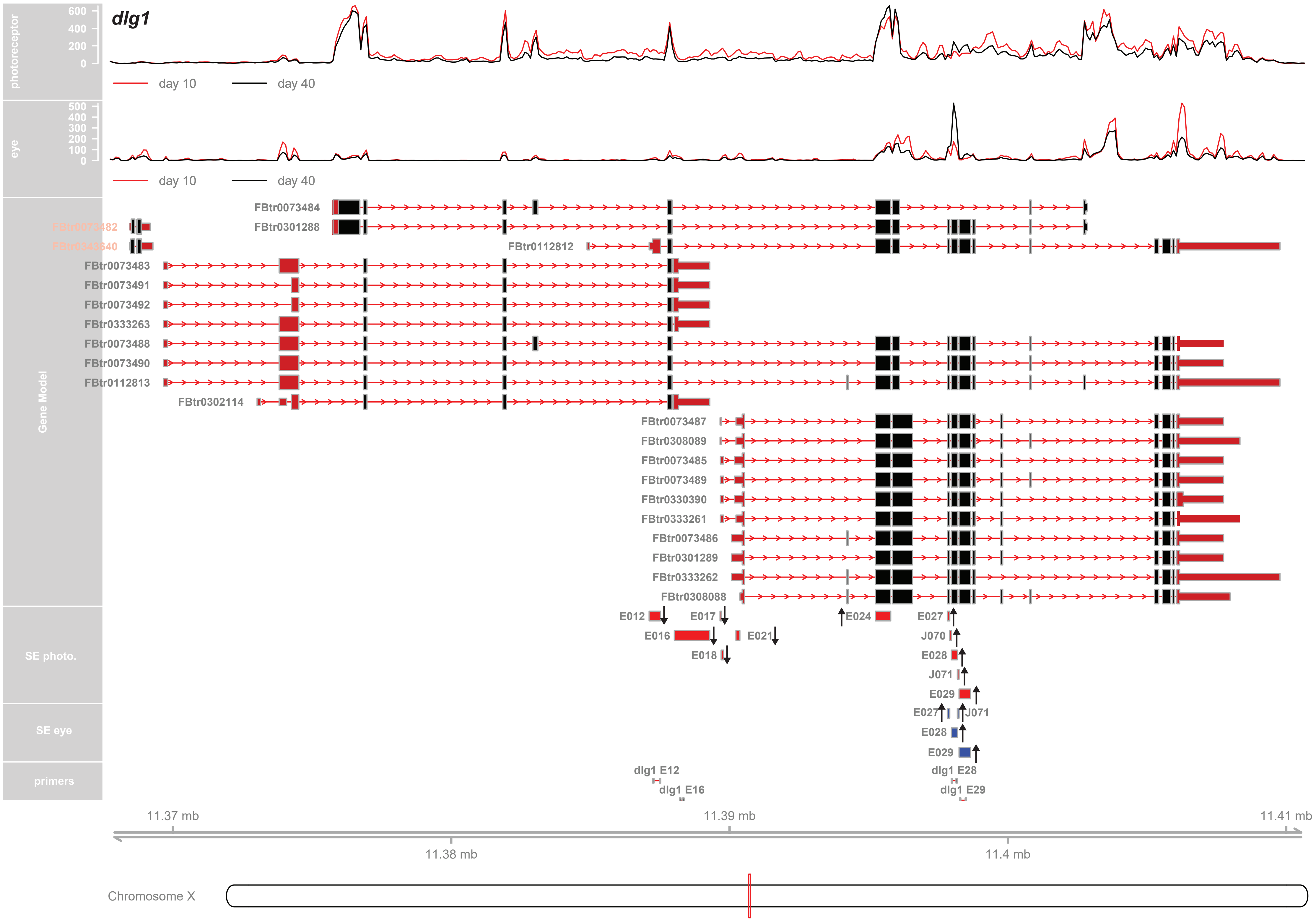
dlg1
photoreceptor
600
400
200
0
day 10
day 40
eye
500
400
300
200
100
0
day 10
day 40
Gene Model
FBtr0073484
FBtr0301288
FBtr0073482
FBtr0343640
FBtr0112812
FBtr0073483
FBtr0073491
FBtr0073492
FBtr0333263
FBtr0073488
FBtr0073490
FBtr0112813
FBtr0302114
FBtr0073487
FBtr0308089
FBtr0073485
FBtr0073489
FBtr0330390
FBtr0333261
FBtr0073486
FBtr0301289
FBtr0333262
FBtr0308088
SE photo.
E012
E017
E024
E027
E016
E021
J070
E018
E028
J071
E029
SE eye
E027
J071
E028
E029
primers
dlg1 E12
dlg1 E16
dlg1 E28
dlg1 E29
11.37 mb
11.39 mb
11.41 mb
11.38 mb
11.4 mb
Chromosome X

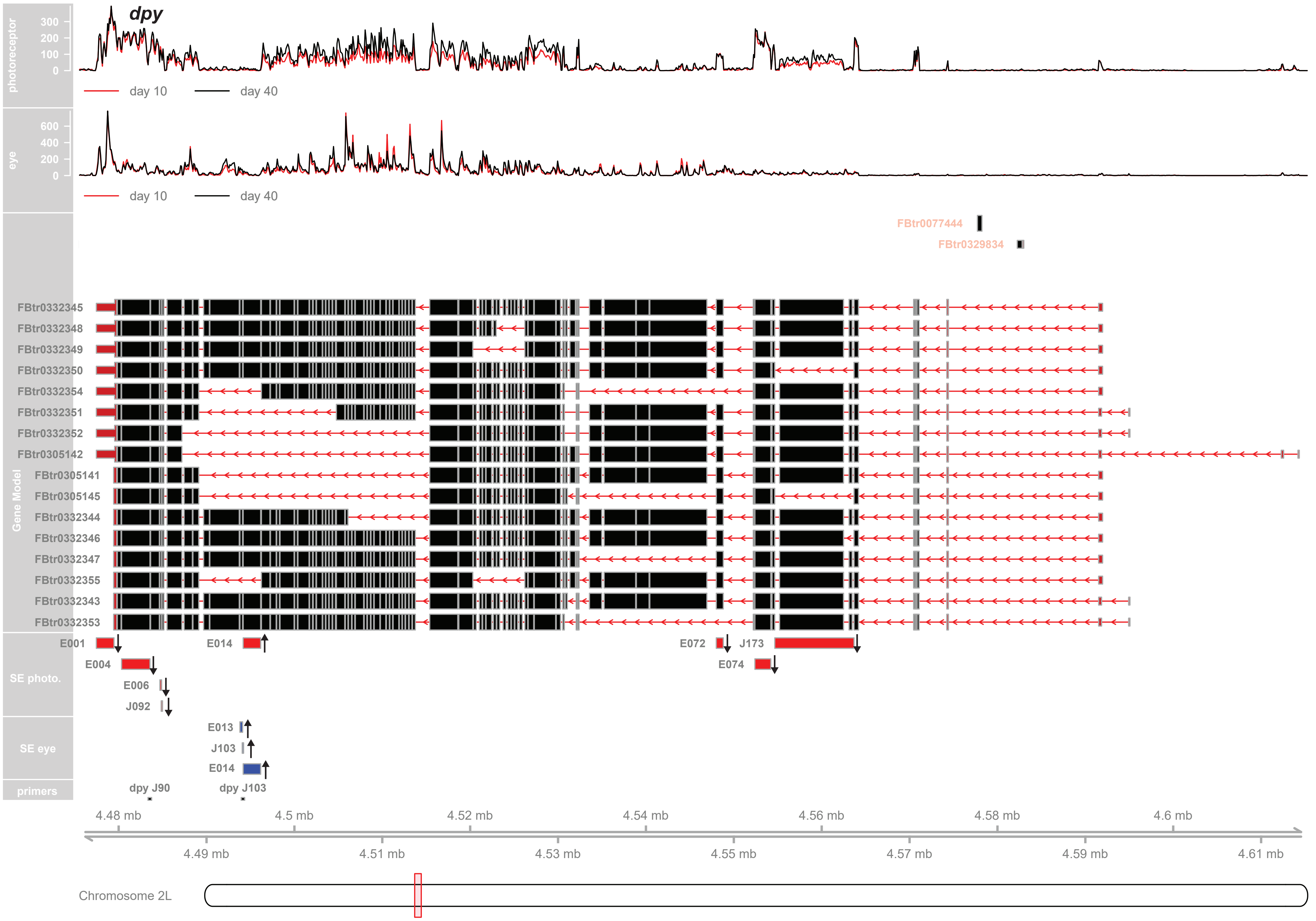
dpy
photoreceptor
300
200
100
0
day 10
day 40
eye
600
400
200
0
day 10
day 40
FBtr0077444
FBtr0329834
Gene Model
FBtr0332345
FBtr0332348
FBtr0332349
FBtr0332350
FBtr0332354
FBtr0332351
FBtr0332352
FBtr0305142
FBtr0305141
FBtr0305145
FBtr0332344
FBtr0332346
FBtr0332347
FBtr0332355
FBtr0332343
FBtr0332353
SE photo.
E001
E014
E072
J173
E004
E074
E006
J092
SE eye
E013
J103
E014
primers
dpy J90
dpy J103
4.48 mb
4.5 mb
4.52 mb
4.54 mb
4.56 mb
4.58 mb
4.6 mb
4.49 mb
4.51 mb
4.53 mb
4.55 mb
4.57 mb
4.59 mb
4.61 mb
Chromosome 2L

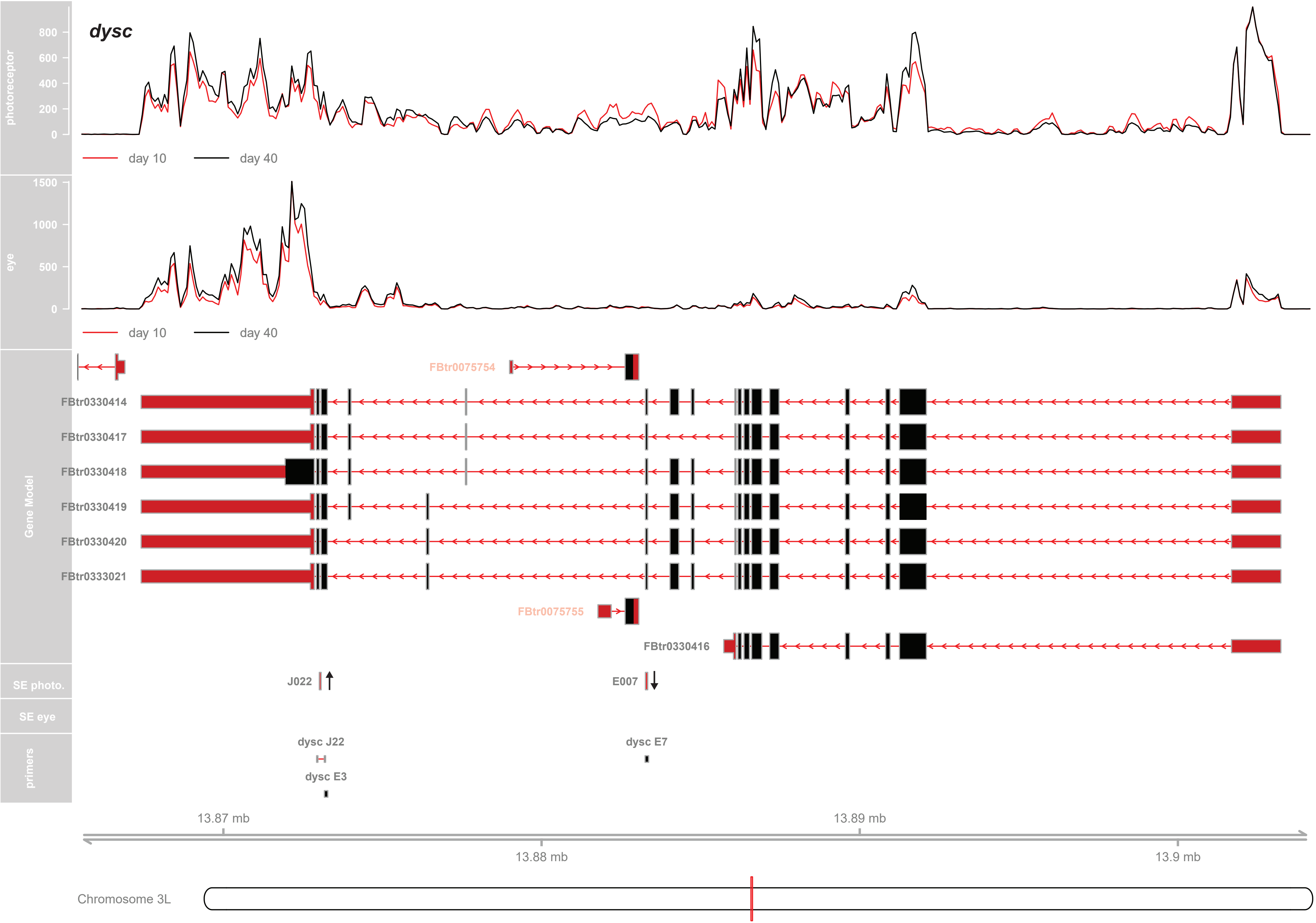

dysc
photoreceptor
800
600
400
200
0
day 10
day 40
eye
1500
1000
500
0
day 10
day 40
Gene Model
FBtr0075754
FBtr0330414
FBtr0330417
FBtr0330418
FBtr0330419
FBtr0330420
FBtr0333021
FBtr0075755
FBtr0330416
SE photo.
J022
E007
SE eye
primers
dysc J22
dysc E7
dysc E3
13.87 mb
13.88 mb
13.89 mb
13.9 mb
Chromosome 3L

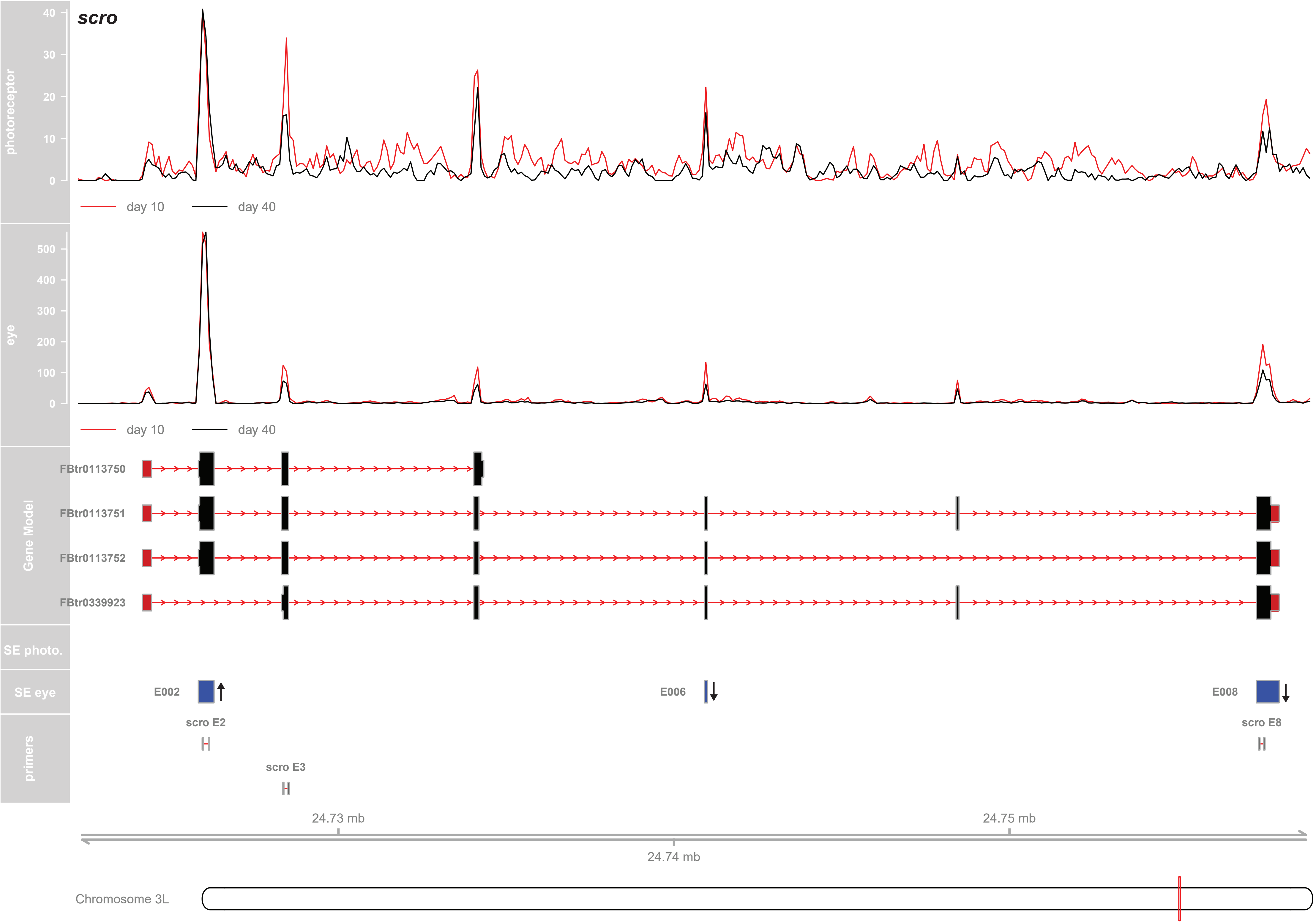
scro
photoreceptor
40
30
20
10
0
day 10
day 40
eye
500
400
300
200
100
0
day 10
day 40
Gene Model
FBtr0113750
FBtr0113751
FBtr0113752
FBtr0339923
SE photo.
SE eye
E002
E006
E008
primers
scro E2
scro E3
scro E8
24.73 mb
24.74 mb
24.75 mb
Chromosome 3L

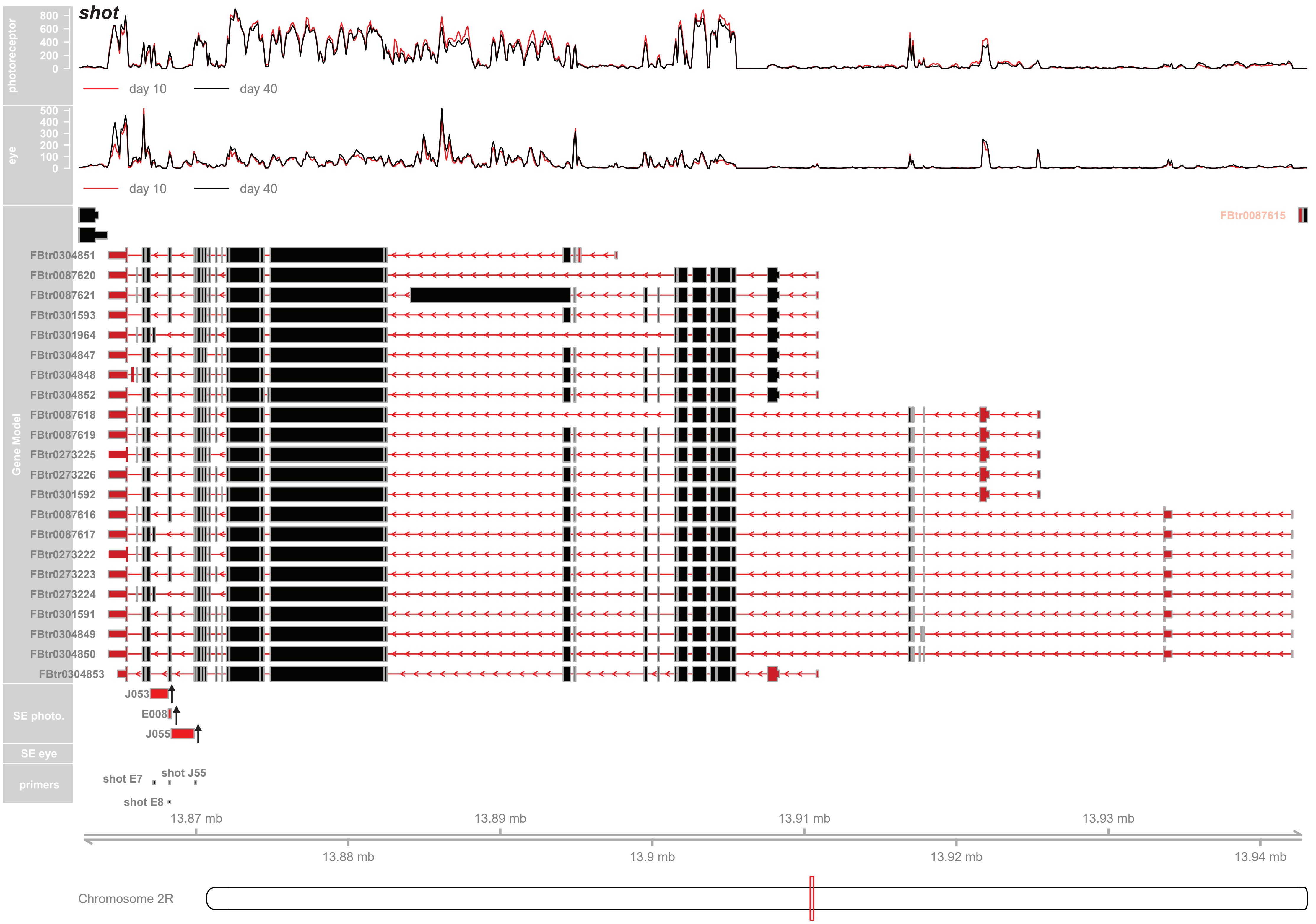

shot
photoreceptor
800
600
400
200
0
day 10
day 40
eye
500
400
300
200
100
0
day 10
day 40
Gene Model
FBtr0087615
FBtr0304851
FBtr0087620
FBtr0087621
FBtr0301593
FBtr0301964
FBtr0304847
FBtr0304848
FBtr0304852
FBtr0087618
FBtr0087619
FBtr0273225
FBtr0273226
FBtr0301592
FBtr0087616
FBtr0087617
FBtr0273222
FBtr0273223
FBtr0273224
FBtr0301591
FBtr0304849
FBtr0304850
FBtr0304853
SE photo.
J053
E008
J055
SE eye
primers
shot E7
shot J55
shot E8
13.87 mb
13.89 mb
13.91 mb
13.93 mb
13.88 mb
13.9 mb
13.92 mb
13.94 mb
Chromosome 2R

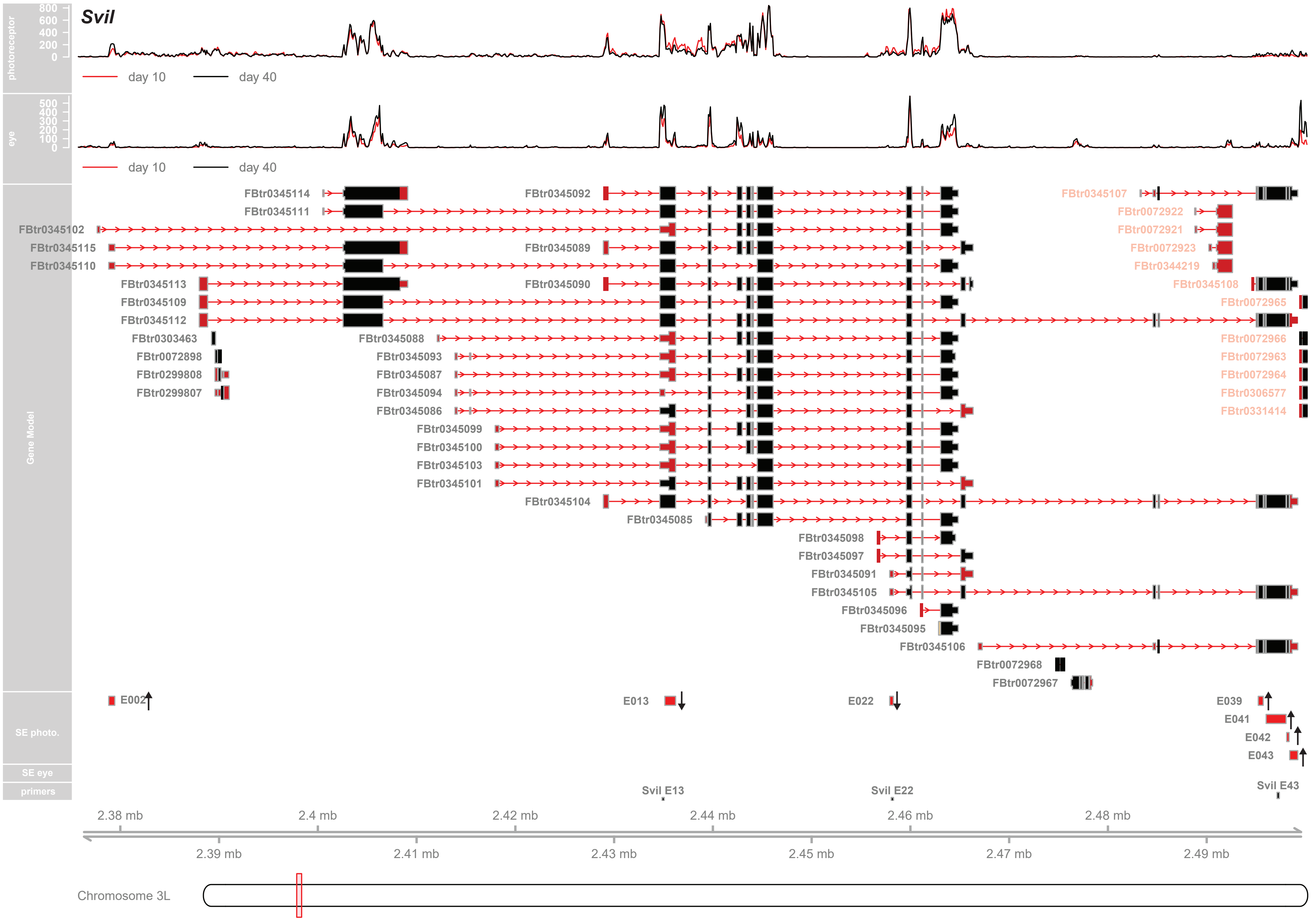
Svil
photoreceptor
800
600
400
200
0
day 10
day 40
eye
500
400
300
200
100
0
day 10
day 40
Gene Model
FBtr0345114
FBtr0345111
FBtr0345102
FBtr0345115
FBtr0345110
FBtr0345113
FBtr0345109
FBtr0345112
FBtr0303463
FBtr0072898
FBtr0299808
FBtr0299807
FBtr0345092
FBtr0345089
FBtr0345090
FBtr0345088
FBtr0345093
FBtr0345087
FBtr0345094
FBtr0345086
FBtr0345099
FBtr0345100
FBtr0345103
FBtr0345101
FBtr0345104
FBtr0345085
FBtr0345098
FBtr0345097
FBtr0345091
FBtr0345105
FBtr0345096
FBtr0345095
FBtr0345106
FBtr0072968
FBtr0072967
FBtr0345107
FBtr0072922
FBtr0072921
FBtr0072923
FBtr0344219
FBtr0345108
FBtr0072965
FBtr0072966
FBtr0072963
FBtr0072964
FBtr0306577
FBtr0331414
SE photo.
E002
E013
E022
E039
E041
E042
E043
SE eye
primers
Svil E13
Svil E22
Svil E43
2.38 mb
2.4 mb
2.42 mb
2.44 mb
2.46 mb
2.48 mb
2.39 mb
2.41 mb
2.43 mb
2.45 mb
2.47 mb
2.49 mb
Chromosome 3L

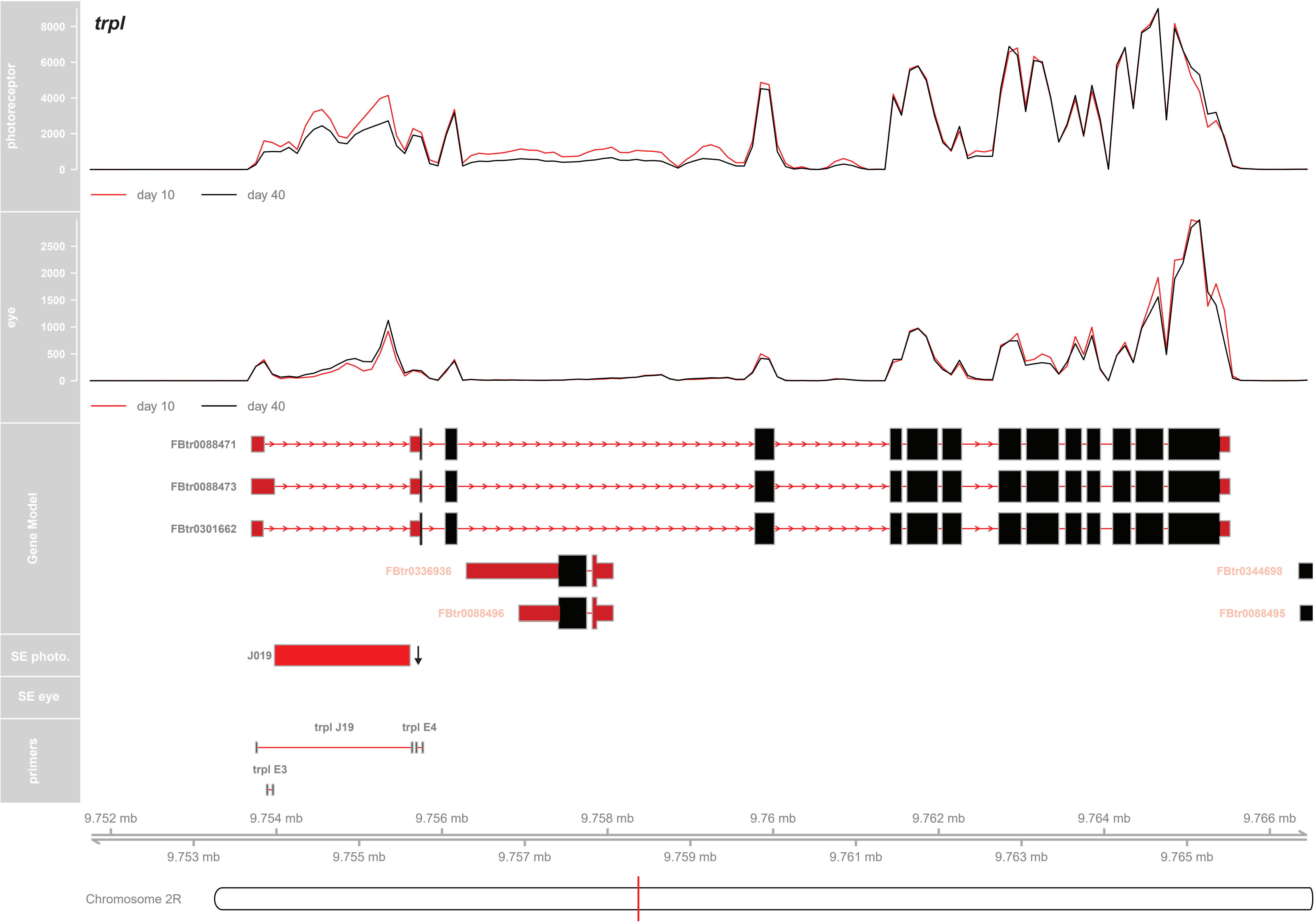
trpl
photoreceptor
8000
6000
4000
2000
0
day 10
day 40
eye
2500
2000
1500
1000
500
0
day 10
day 40
Gene Model
FBtr0088471
FBtr0088473
FBtr0301662
FBtr0336936
FBtr0344698
FBtr0088496
FBtr0088495
SE photo.
J019
SE eye
primers
trpl J19
trpl E4
trpl E3
9.752 mb
9.754 mb
9.756 mb
9.758 mb
9.76 mb
9.762 mb
9.764 mb
9.766 mb
9.753 mb
9.755 mb
9.757 mb
9.759 mb
9.761 mb
9.763 mb
9.765 mb
Chromosome 2R

Stegeman_FigS4

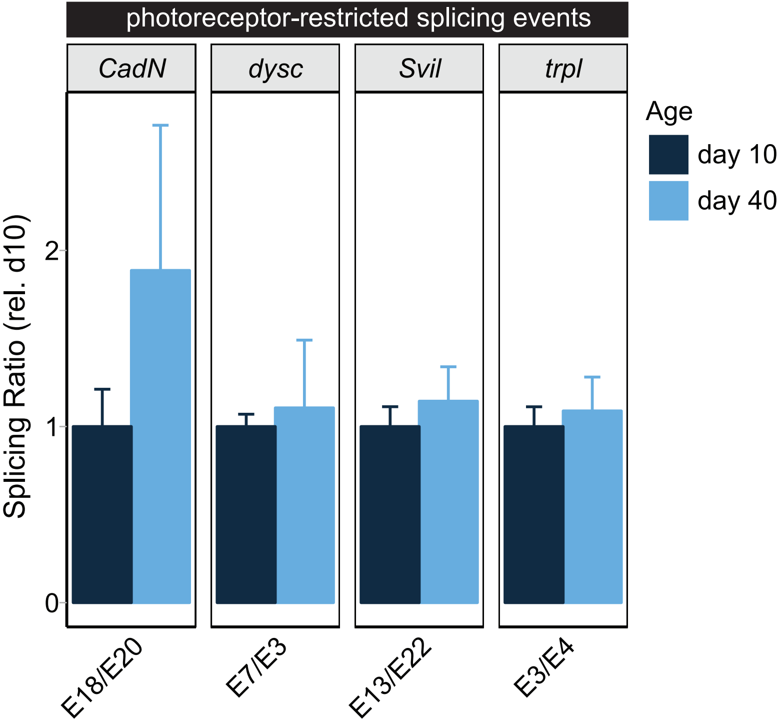

Stegeman_FigS5

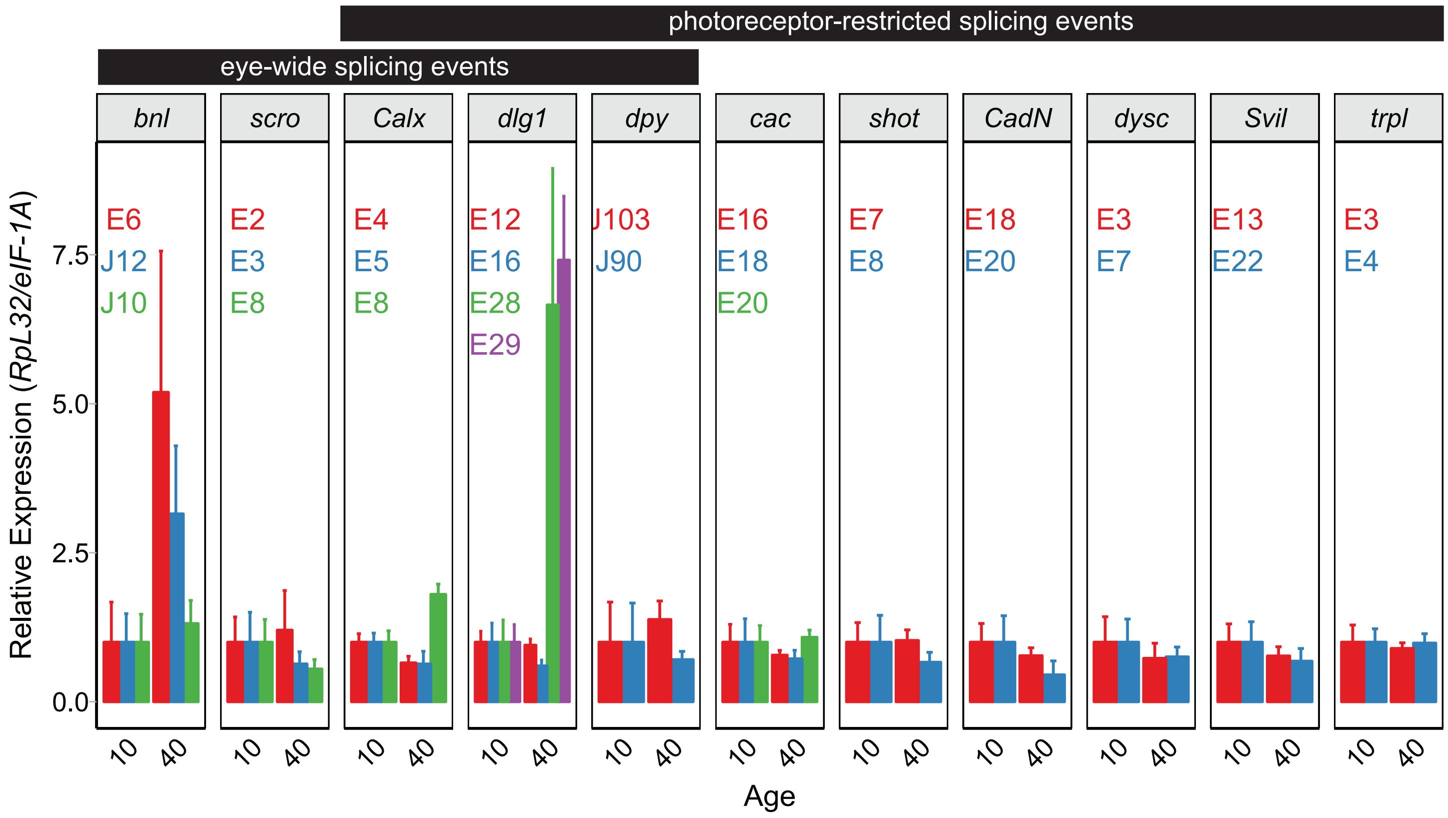

Supplement: Supplementary file 1 [file ACEL-17-e12817-s001.pdf]
